# PepQueryMHC: rapid and comprehensive tumor antigen prioritization from immunopeptidomics data

**DOI:** 10.1186/s13059-025-03923-w

**Published:** 2025-12-23

**Authors:** Seunghyuk Choi, Bing Zhang

**Affiliations:** 1 Lester and Sue Smith Breast Center, Baylor College of Medicine, 1 Baylor Plaza, Houston, TX 77030 USA; 2 Department of Molecular and Human Genetics, Baylor College of Medicine, Houston, TX 77030 USA; 3 School of Software, College of Computer Science, Kookmin University, Seoul, 02707 Republic of Korea

**Keywords:** Immunopeptidome, Tumor antigens, Immunoinformatics, Proteogenomics, Major histocompatibility complex

## Abstract

Determining tumor-specificity of MHC-bound peptides is crucial for cancer immunotherapy development, yet current methods struggle with class II peptides and non-reference sequences. We introduce PepQueryMHC, an ultra-fast tool that integrates MHC-bound peptide sequences with translated RNA-seq reads for efficient tumor antigen prioritization. We demonstrate its versatility in prioritizing class I and II tumor antigens, mapping the cellular origins of presented peptides, and resolving uncertainties surrounding the prevalence of proteasome-spliced peptides.

**Supplementary Information:**

The online version contains supplementary material available at 10.1186/s13059-025-03923-w.

## Background

T cell-mediated immunity plays a pivotal role in the adaptive immune system, eliminating infected or cancerous cells through the recognition of specific antigens. This immune surveillance relies on the presentation of major histocompatibility complex (MHC)-bound peptides (pMHCs) on the cell surface [1]. Leveraging this mechanism, cancer immunotherapy has emerged as a major breakthrough by targeting tumor-specific pMHCs, including tumor-specific antigens (TSAs) and tumor-associated antigens (TAAs). These peptides, arising from mutations, fusions, aberrant gene expression, dysregulated splicing, and abnormal translation from presumed non-coding regions, serve as promising targets for immunotherapy [2, 3]. Therefore, comprehensive identification of tumor-specific pMHCs is crucial for developing cancer immunotherapy.

Advances in mass spectrometry (MS)-based immunopeptidomics, de novo MS/MS sequencing, and computational proteogenomics have transformed pMHC characterization, enabling systematic profiling of both class I and class II immunopeptidomes [4–8]. Unlike earlier approaches that relied primarily on DNA or RNA sequencing to predict neoantigens from somatic mutations [9–11], immunopeptidomics allows for direct identification of pMHCs from individual samples, including hundreds to thousands derived from non-reference peptides—those absent from the reference human proteome. For example, by integrating de novo MS/MS sequencing and RNA-seq sequencing data, pXg identifies diverse pMHCs, including both wild-type and mutated peptides derived from protein coding genes, frameshifts, non-coding RNAs, untranslated regions, alternative splicing events, antisense RNAs and viral proteins. Alongside methodological advancements, several repositories of MS-identified pMHCs have expanded the pool of putative tumor antigen candidates, encompassing reference, non-reference or post-translationally modified peptides across various tumor types and normal tissues [12–15]. However, as the repertoire of pMHCs identified from cancer samples expands, distinguishing truly tumor-specific peptides becomes increasingly challenging, highlighting the need for effective prioritization strategies.

Omics data from tumor and normal samples are widely used to assess the tumor-specificity of pMHCs. DNA sequencing enables neoantigen prioritization by linking peptides to somatic mutations [9, 11]. For non-mutated pMHCs, immunopeptidome or proteome data from normal tissues, such as those available in the HLA Ligand Atlas [12] and Human Protein Atlas [16] can provide useful context. However, the absence of detection in these datasets does not guarantee tumor-specificity, given the limited sensitivity of mass spectrometry, especially for low-abundance peptides, and the restricted number and diversity of normal tissue samples. In contrast, RNA-seq offers a more sensitive, albeit indirect, means of inferring peptide presence based on transcript abundance. Although imperfect, this conservative approach helps mitigate the risk of on-target, off-tumor toxicity.

Early RNA-based efforts to prioritize pMHCs relied on mRNA profiling data from The Cancer Genome Atlas (TCGA) [17] and the Genotype-Tissue Expression (GTEx) project [18], enabling rapid assessment of annotated coding genes across large cohorts [19]. However, these approaches were limited to pMHCs derived from aberrantly expressed canonical coding genes. More recently, BamQuery [20] has advanced tumor antigen prioritization by leveraging the STAR aligner [21] to both generate a reference genome index and to map reverse-translated peptide-coding sequences. This approach could incorporate known genetic variants and annotated splice junctions, and estimate local RNA expression based on RNA-seq read coverage over the mapped genomic regions, enabling more in-depth discovery of a wide array of tumor antigens [22]. However, the complexity of reverse translation increases exponentially with peptide length due to the degeneracy of the genetic code, making it feasible only for short MHC-I peptides (8–11 amino acids) but impractical for longer MHC-II peptides, thereby capturing only a partial view of the immunopeptidome. In addition, prioritizing pMHCs derived from unannotated variants, novel splice junctions, or sequences absent from the reference human genome—such as oncogenic viral sequences—remains challenging even for class I peptides, further constraining the scope of tumor antigen prioritization. Moreover, BamQuery, along with other tools such as PGx [23] and ACTG [24], relies heavily on predefined reference gene models and genome sequences, rendering them ill-suited for detecting unannotated transcripts that lie outside the assumed search space. Finally, BamQuery imposes substantial computational demands, requiring over 30 GB of memory for even a single query.

To address these limitations, we introduce PepQueryMHC, a computational tool for ultra-fast, memory efficient comparison of class I and II pMHC sequences against translated RNA-seq reads from matched samples and extensive public tumor and normal datasets. This enables comprehensive tumor antigen prioritization, even for peptides originating from unannotated genomic contexts. We benchmark PepQueryMHC against BamQuery and then demonstrate its versatility in prioritizing class I and II tumor antigens, mapping the cellular origins of presented peptides, and resolving uncertainties surrounding the prevalence of proteasome-spliced peptides.

## Results

### Core algorithm of PepQueryMHC

**Fig. 1:**
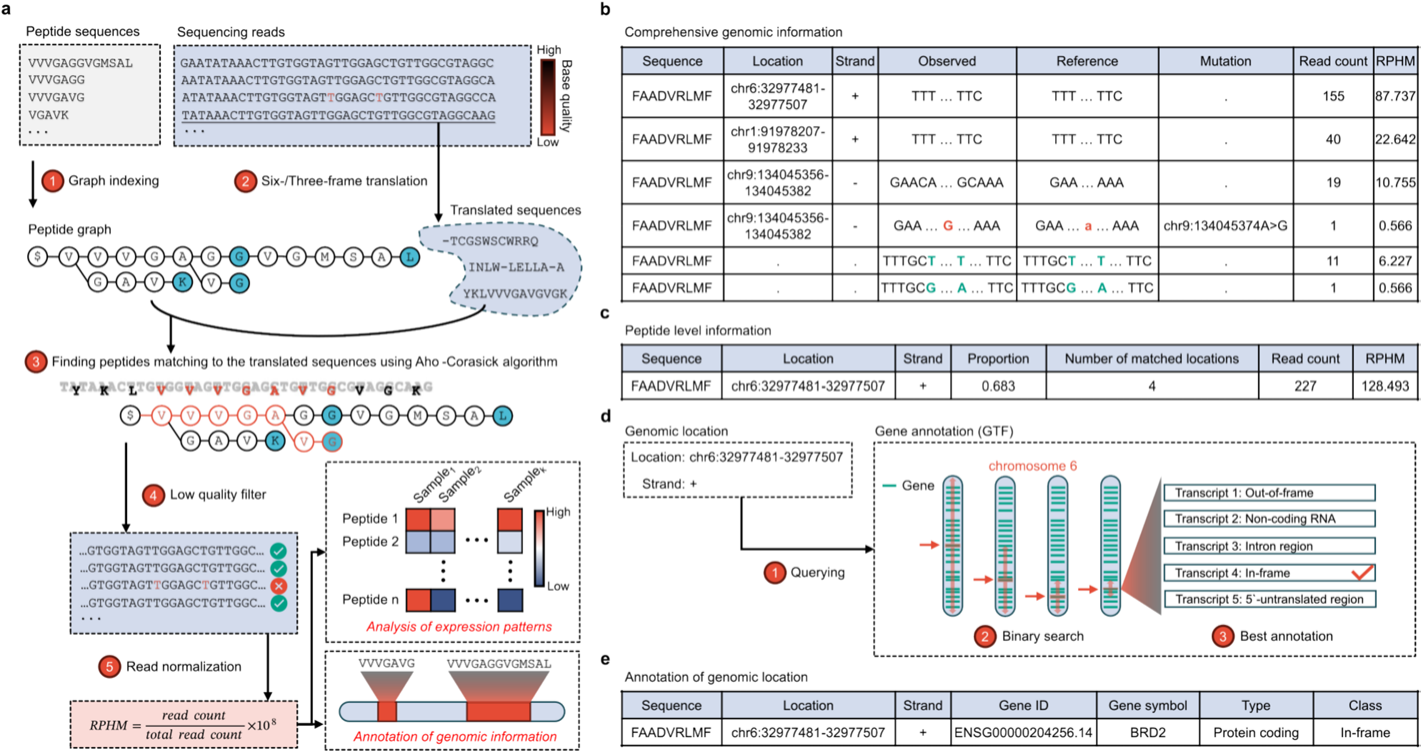
Core algorithm and input/output overview of PepQueryMHC. **a-c** Overview of the peptide-to-read matching algorithm, along with input and output formats. **a** Peptides are represented as a graph data structure using the Aho-Corasick algorithm. Sequencing reads are translated into peptides in parallel, followed by finding peptides matching the translated sequences. Matched reads with low quality are filtered out. The resulting normalized read counts (reads per hundred million, RPHM) and matched genomic regions are used for downstream analysis. **b**,** c** Two types of output summarizing genomic- and peptide-level information. **b** A dot in the Location column indicates that the peptide maps to unmapped reads. A single peptide may align to multiple unmapped sequences. **c** Peptide level output includes the total read count and the genomic region supporting the peptide with the highest abundance. **d**,** e** Algorithm and input/output for genomic annotation. **d** The GTF file is indexed using an interval tree, enabling efficient identification of overlapping genes through binary search. **e** Example of the final output after annotation

PepQueryMHC employs the Aho-Corasick algorithm [25] to construct a trie-based graph encoding peptide suffix and prefix relationships, allowing simultaneous matching of multiple peptides against six- or three-frame translated RNA-seq reads (Fig. 1a, Additional file 1: Fig. S1). This approach eliminates exhaustive pairwise comparisons, enabling peptide-read matching in linear time relative to the total read length. Matches are excluded if the probability of any sequencing error in the aligned region exceeds 0.05 (Additional file 1: Fig. S2), ensuring high-quality results. The number of matched reads per peptide is then normalized to the total read count and scaled by 1 × 10^8^ to yield read per hundred million (RPHM) values. Both raw and normalized read counts are reported together with their matched genomic information, allowing for the estimation of both the quantity and origin of the peptides. To support downstream analysis, two distinct outputs are provided: one at the genomic level (Fig. 1b) and another at the peptide level (Fig. 1c). At the genomic level, comprehensive annotation derived from RNA-seq reads—including both mapped and unmapped reads—is reported, allowing in-depth exploration of the corresponding genomic locations. At the peptide level, an RPHM value is provided for each peptide, calculated by summing RPHM values across all associated genomic locations, thereby reflecting the overall RNA expression contributing to the peptide. Additionally, the output also highlights the genomic location with the highest expression.

To consistently classify peptide types at each genomic location, the tool takes genomic coordinates, strand information, and a genomic annotation file (GTF) as input. The GTF file is indexed using an interval tree structure, allowing efficient gene identification within a given genomic location using binary search (Fig. 1d, e). In addition to identifying all matched reads (regardless of genomic regions) for each peptide, PepQueryMHC also supports targeted evaluation, which counts reads in a specific region of interest. This feature is particularly useful for quickly querying a small number of peptides (< 1,000) with well-characterized genomic information.

## Performance benchmarking

**Fig. 2:**
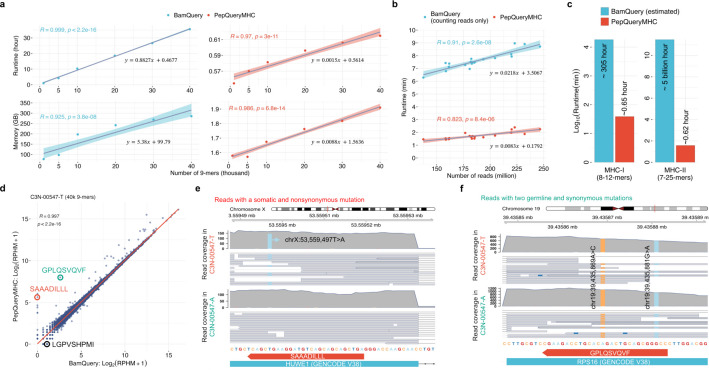
Performance benchmarking of PepQueryMHC versus BamQuery.** a** Runtime and memory usage of PepQueryMHC and BamQuery for varying numbers of randomly sampled pMHC-I 9-mers (mean of three replicates each). **b** Runtime assessment according to the number of reads, using a fixed input of 10,000 9-mers from (**a**). For BamQuery, only the read-counting step was measured, excluding preprocessing. **c** Runtime comparison for complete pMHC-I and pMHC-II datasets, with BamQuery runtimes estimated using linear regression.** d** Comparison of RPHM values for peptides quantified by both tools. **a**, **b**, **d** Pearson correlation coefficients (R) and corresponding *p*-values are indicated. **e**,** f** Representative examples of peptides derived from a somatic/nonsynonymous mutation (**e**) and from two germline/synonymous mutations (**f**)

We benchmarked PepQueryMHC against the state-of-the-art BamQuery using 72,885 pMHC-I (8–12-mers) and 31,526 pMHC-II (7–25-mers) peptides from a prior immunopeptidomics study on ten lung adenocarcinoma (LUAD) tumors [5], along with RNA-seq data from matched tumors and adjacent normal tissues (Additional file 1: Fig. S3, Additional file 2) [26]. To assess runtime and memory usage, we randomly sampled 1000, 5000, 10,000, 20,000, 30,000 and 40,000 peptides from the 45,349 9-mers in the pMHC-I dataset and matched them to all RNA-seq reads using both tools. Although both tools showed linear increases in runtime and memory usage with the number of input peptides, PepQueryMHC exhibited a substantially lower slope, indicating minimal computational overhead as input size grows (Fig. 2a). As a result, PepQueryMHC was 1.79 to 57.90 times faster and 49.25 to 149.41 times more memory-efficient than BamQuery as input peptide number increased (Additional file 3).

We further examined runtime as a function of RNA-seq read depth using the 20 BAM files with varying read counts (Fig. 2b). BamQuery used approximately 80% of its total runtime for preprocessing steps, such as reverse translation and STAR alignment, resulting in substantially slower overall performance compared to PepQueryMHC. Since BamQuery performs preprocessing once per batch, we also evaluated its runtime excluding preprocessing time to assess the effect of input read count. Runtime per sample was averaged across three runs using 10,000 9-mers from the pMHC-I dataset. For both methods, runtime increased with the number of reads in the BAM file. Notably, even when considering only the read counting step, PepQueryMHC consistently outperformed BamQuery. Moreover, BamQuery’s runtime slope was 2.63 times steeper than that of PepQueryMHC, underscoring the superior scalability of PepQueryMHC across varying batch sizes.

To assess performance in a setting reflective of a real-world application, we queried the full pMHC-I and pMHC-II datasets (72,885 and 31,526 peptides, respectively) against the 20 matched BAM files. PepQueryMHC completed both analyses in under 40 min. In contrast, BamQuery was unable to complete either analysis within a reasonable timeframe, with estimated runtimes of 305 h for the class I dataset and over 5 billion hours for class II (Fig. 2c, Additional file 1: Fig. S4). This prohibitive runtime for class II analysis arises because BamQuery was originally designed for short peptides (8–12-mers), making its algorithm infeasible for processing longer class II peptides (typically 13–17-mers).

To compare quantifications between the two tools, we analyzed 40,000 9-mers and observed highly correlated RNA expression estimates across tumors (*R* = 0.996–0.998, *p* < 2.2e^−16^, Additional file 1: Fig. S5). However, off-diagonal peptides in the scatterplots revealed discrepancies. Higher counts reported by PepQueryMHC, such as for the neopeptide SAAADILLL and the multi-location peptide GLPQSVQVF in C3N-00547-T (Fig. 2d), resulted from its ability to map reads containing sample-specific sequences. In the first case, PepQueryMHC successfully aligned the neopeptide, which BamQuery failed to map (Fig. 2e). In the second case, both tools aligned the peptide to the wild-type sequence at the ZNF90 locus (Additional file 1: Fig. S6a, respectively), but only PepQueryMHC additionally aligned it to the RPS16 locus by accounting for two synonymous variants (Fig. 2f). These differences arise because BamQuery relies on variants annotated in dbSNP and thus cannot align reads containing sample-specific somatic mutations absent from this reference. In contrast, PepQueryMHC directly matches against all reads, allowing detection of sample-specific sequences beyond known dbSNP variants. Overall, 1.8–5.4% of the reads matched by PepQueryMHC did not align exactly to the reference genome, including 1.3–2.8% that mapped with variants and the rest that were unmappable likely due to highly polymorphic alleles (e.g., HLA) or non-human sequences (Additional file 1: Fig. S6b). Conversely, lower counts reported by PepQueryMHC, such as for LGPVSHPMI (Fig. 2d), were due to the removal of low-quality reads (Additional file 1: Fig. S2). Thus, PepQueryMHC is more efficient and enables comprehensive, robust quantification.

## Prioritizing class I and II tumor antigens

To demonstrate the utility of PepQueryMHC in prioritizing tumor antigens, we queried all peptides in the LUAD class I and class II datasets against the 20 BAM files from matched tumors and adjacent normal tissues, 2,403 BAM files from 52 normal human tissue types in GTEx [18], and eight human mTEC BAM files [27, 28]. We established a rigorous prioritization strategy integrating RNA- and protein-level tumor specificity while accounting for immune tolerance (Additional file 1: Fig. S7a). To define thresholds for negligible RNA expression, we calculated RPHM values for each peptide across 2,403 normal human tissue samples and averaged values across samples with non-zero expression. Peptides were then stratified by length and MHC class, and the 1 st percentile of average RPHM values within each group was used as the threshold. This approach yielded threshold values of approximately 2 RPHM across all stratified groups (Additional file 1: Fig. S7b, c).

We first evaluated our prioritization strategy using 9,169 viral and 223,242 normal pMHCs. Among them, 9,109 viral pMHCs (99.35%) were retained as virus-specific, demonstrating the high sensitivity of our approach. In contrast, 222,562 normal pMHCs (99.70%) were excluded, indicating that the majority of normal pMHCs can be effectively filtered out using this strategy. To further enhance specificity, we incorporated data from the Human Protein Atlas [16] to exclude pMHCs with protein-level evidence, providing complementary validation based on peptide expression.

**Fig. 3:**
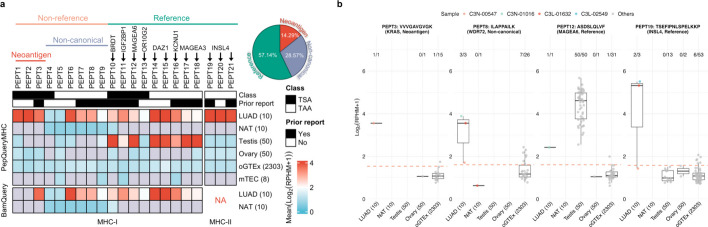
Application of PepQueryMHC in tumor antigen prioritization.** a** Tumor-specific antigens (TSAs) and tumor-associated antigens (TAAs) prioritized from a LUAD immunopeptidomics study. LUAD, NAT, oGTEx, and mTEC refer to lung adenocarcinoma, normal adjacent tissue, other GTEx (excluding testis and ovary), and medullary thymic epithelial cell, respectively. The Report row annotates previously reported tumor antigens. **b** Representative examples of prioritized tumor antigens, including a class I neoantigen (KRAS 12G > D), a class I peptide from a non-canonical region (WDR72), a class I peptide from a canonical cancer/testis antigen (MAGEA6), and a class II peptide derived from a canonical gene (INSL4). The defined thresholds are shown as a red dash line per each peptide. Each value represents the number of samples exceeding the threshold out of the total samples (*e.g.*, 1/15, 7/25)

Using this prioritization strategy, we identified 21 prioritized tumor antigens including 18 class I peptides—classified into eight tumor-specific antigens (TSAs) and 10 tumor-associated antigens (TAAs)—and three class II peptides, all of which were TAAs (Fig. 3a, Additional file 1: Fig. S8, 9, Additional file 4). While reference peptides and neoantigens accounted for 15 out of the 21 (71.43%) of the prioritized antigens, non-canonical regions were significantly enriched for tumor antigens relative to their overall representation across peptide categories (Fisher’s exact test *p* = 1.039e^−6^). BamQuery failed to detect 24% of these prioritized antigens, including two class I neoantigens and the three class II TAAs.

Among the class I peptides prioritized by PepQueryMHC, nine were non-reference peptides, comprising three neoantigens derived from *CIC*, *HUWE1*, and *KRAS*, respectively, and six non-canonical antigens. For example, RNA expression exceeding the defined thresholds was observed in only 0.04% of GTEx normal samples (excluding testis and ovary, hereafter referred to as oGTEx) for the KRAS (12G > V) mutant peptide and 0.30% for a non-canonical WDR72 sequence (Fig. 3b). The remaining nine class I peptides were reference peptides, eight of which originated from six genes (MAGEA3, MAGEA6, BRDT, DAZ1, KCNU1, and IGF2BP1) that are expressed in testis but absent from oGTEx and mTECs, with some already known as cancer/testis (C/T) antigens [29–31]. For example, a peptide derived from the well-established C/T antigen MAGEA6 (ASDSLQLVF) showed recurrent mRNA expression across all testis samples in GTEx, while only 0.04% of the oGTEx samples exhibited non-negligible mRNA expression (Fig. 3b). In addition, IGF2BP1 is upregulated in most cancers and retains strong oncogenic potential, highlighting its promise as an immunotherapy target [32, 33]. Notably, to our knowledge, KCNU1 has not previously been reported as a tumor antigen, representing a new candidate for further investigation as a potential immunotherapeutic target. Finally, one class I peptide from OR10G2 showed minimal expression across all normal tissues, including testis and ovary.

For class II peptides, PepQueryMHC prioritized three peptides derived from the same gene, INSL4, which were consistently detected in three LUAD patients—two of whom exhibited high expression. RNA expression exceeding the defined threshold was observed in only 0.26% of oGTEx samples for these peptides, supporting their potential as tumor-associated antigens.

Among the 21 prioritized tumor antigens, 12 had been reported in previous class I or II immunopeptidomics studies [13–15] (Fig. 3a). Notably, PEPT7 and PEPT8 originated from a non-coding transcript of *WDR72*, and non-canonical pMHCs derived from *WDR72* have been recurrently observed in glioblastoma, melanoma, and lung cancer samples in IEAtlas-cancer, highlighting their potential as shared TAAs. Importantly, none of the 21 were found in normal immunopeptidome databases such as the HLA Ligand Atlas [12] or IEAtlas-normal [13], supporting their tumor specificity.

## Mapping the cellular origins of presented peptides

**Fig. 4:**
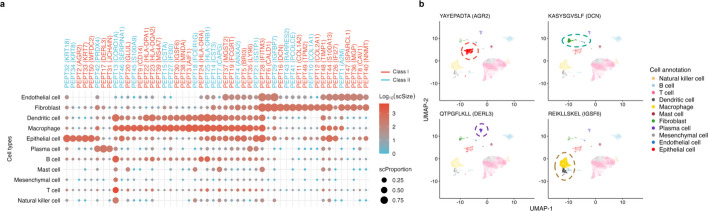
Application of PepQueryMHC in cellular origin mapping using single-cell RNA-seq dataset** a** Top 50 peptides with the highest expression variability across cell types. scSize represents the number of cells expressing each peptide, while scProportion indicates the proportion of expressing cells within each cell type. **b** UMAP plot of all cells from a LUAD single-cell RNA-seq dataset, with cells expressing selected cell type-specific peptides highlighted

Bulk tumor immunopeptidomics captures peptides presented by a mixture of cell types, making it crucial to determine their cellular origin to gain biological insights and guide clinical applications. To this end, we used PepQueryMHC to query all pMHC sequences from the LUAD immunopeptidomics study against RNA-seq reads from eight primary tumors in an independent LUAD single-cell RNA-seq study [34] (Additional file 1: Fig. S10). Despite limited sequencing depth, the analysis identified numerous peptides with distinct cell type-specific expression patterns. Figure 4a shows the top 50 class I and class II peptides with the highest cell type specificity, demonstrating the feasibility of mapping both peptide classes to single-cell RNA-seq reads (Additional file 5). The largest group of peptides showed enriched expression in macrophages and dendritic cells, including those derived from *HLA-DPA1*, *HLA-DQA2*, *HLA-DRA*, and *HLA-DRB1*, genes encoding the α and β chains of MHC class II molecules that are highly restricted to professional antigen-presenting cells. The second major cluster comprised peptides whose source genes were enriched in fibroblasts, with partial expression overlap in endothelial cells. Examples included *COL1A1*, *TIMP1*, *SPARCL1*, and *NNMT*, indicating contributions from stromal components of the tumor microenvironment. This analysis also revealed several peptides originating from genes enriched in epithelial cells, including *KRT18*, *KRT8*, *AGR2*, *KRT7*, and *WFDC2*. To illustrate cell type-specific expression patterns, we generated UMAP plots for selected peptides with strong specificity, including an epithelial specific peptide from *AGR2*, a fibroblast-specific peptide from *DCN*, a plasma cell specific peptide from *DERL3*, and a dendritic cell and macrophage-specific peptide from *ISGF6* (Fig. 4b). The utility of this analysis is expected to improve with increased sequencing depth in single-cell RNA-seq.

### Reassessing previously classified spliced peptides

**Fig. 5:**
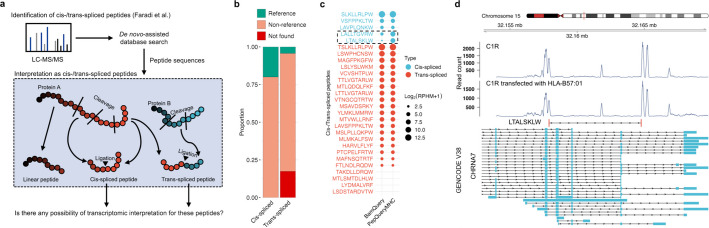
Application of PepQueryMHC in reannotation of previously reported cis-/trans-spliced peptides. **a** Overview of the computational pipeline developed by Faradi et al. for identifying proteasome-spliced peptides.** b** Proportion of previously reported cis-/trans-spliced pMHC-I sequences mapping to RNA-seq reads from matched samples. **c** RPHM values of each peptide, with peptides showing quantification differences between PepQueryMHC and BamQuery highlighted by a dotted box. **d** Example of a cis-spliced pMHC-I sequence mapping to a novel antisense RNA, supported by RNA-seq reads in two samples

Faradi and colleagues previously identified numerous proteasome-spliced peptides as potential tumor antigens using a de novo-assisted database search strategy [35]. They classified high-quality de novo peptides into three classes including linear, cis-spliced, and trans-spliced peptides, when possible (Fig. 5a). However, the reported prevalence of spliced peptides remains controversial [36], and further investigation is warranted.

To investigate whether these peptides could instead originate from RNA-derived products, we re-examined 28 high-confidence, experimentally confirmed pMHC-I peptides previously attributed to cis- or trans-proteome splicing [35] by querying matched RNA-seq data using both PepQueryMHC and BamQuery (Additional file 6). Notably, transcriptomic evidence was found for 24 (85.71%) of these peptides by both methods, with 22 mapping to non-canonical regions (Fig. 5b, Additional file 1: Fig. S11). Two peptides with the highest RNA abundance (> 7400 RPHM)—TSIKLLRLPW and SIKLLRLPW—were mapped to the same in-frame sequence within the ENO1 gene, sharing identical genomic coordinates. These peptides are highly similar, with the shorter entirely contained within the longer. They were previously annotated as trans-spliced and cis-spliced peptides, respectively.

PepQueryMHC and BamQuery identified the same number of genomic locations for most peptides, resulting in concordant mappings in 22 out of 24 peptides (91.67%, Fig. 5c). However, PepQueryMHC detected more highly expressed genomic loci for two peptides (LALLTGVRW and LTALSKLW), likely due to its enhanced ability to capture unannotated transcripts. For instance, BamQuery annotated chr2:64,522,438–64,522,461 as the most abundant genomic location for the peptide LTALSKLW, with an RPHM value of 2.09. In contrast, PepQueryMHC identified a novel isoform within the CHRNA7 gene that encoded the same peptide with a substantially higher RPHM of 7.06 (Fig. 5d).

As positive controls, we evaluated five well-characterized spliced peptides—NTYASPRFK, RTKQLYPEW, RSYVPLAHR, SLPRGTSTPK, and IYMNGTANFSF—whose proteasome-mediated splicing mechanisms have been extensively validated [37–41]. Because these peptides were originally identified from kidney cancer, melanoma, or Epstein-Barr virus (EBV)-transformed B cells, we queried them against RNA-seq data from the A-498 (kidney cancer), A-375 (melanoma) and B-LCL (EBV-transformed B cell) lines [42, 43]. Notably, PepQueryMHC detected no transcriptomic evidence for these peptides in our analysis, supporting the notion that they may indeed arise from proteasome-mediated splicing events. In contrast, many peptides previously classified as proteasome-spliced could be explained by alternative transcriptomic origins, challenging the reported prevalence of proteome splicing and demonstrating the superior sensitivity of PepQueryMHC in detecting RNA-derived sequences.

## Discussion

Accurate identification and prioritization of tumor-specific pMHCs is a critical step in cancer immunotherapy development. While mass spectrometry–based immunopeptidomics has vastly expanded our ability to characterize the MHC-bound peptidome, distinguishing truly tumor-specific antigens from self-peptides remains difficult, especially for non-reference peptides absent from the canonical proteome. In this study, we introduce PepQueryMHC, a fast, memory-efficient tool that addresses these challenges by enabling comprehensive and scalable peptide-to-transcriptome mapping for both MHC class I and class II pMHCs.

Benchmarking against the current state-of-the-art, BamQuery, demonstrated that PepQueryMHC offers orders-of-magnitude improvements in speed and memory usage while maintaining high concordance in RNA expression quantification. These computational gains are critical for real-world applications involving large immunopeptidomics datasets, especially for class II pMHCs and non-reference peptides, which are challenging for reverse-translation-based methods due to increased degeneracy and computational complexity. Moreover, PepQueryMHC’s capacity to detect transcriptomic evidence even in the presence of variants or unannotated transcript isoforms improves mapping exhaustiveness and reduces false negatives—a notable limitation of existing tools.

Using PepQueryMHC, we established a rigorous tumor antigen prioritization framework that incorporates RNA expression across tumors, normal tissues, and mTECs, enhancing confidence in tumor specificity and immunogenic potential. Notably, 21 tumor antigens were prioritized from LUAD datasets, including both neoantigens and non-canonical antigens, many of which were undetectable by BamQuery. Among these were known cancer-testis antigens, previously reported TAAs, and novel candidates such as KCNU1 and OR10G2, which merit further functional validation. Importantly, none of the prioritized peptides were detected in normal HLA ligandome databases, supporting their tumor selectivity and translational potential.

In addition to tumor-normal comparisons, we leveraged single-cell RNA-seq data to infer the cellular origins of presented peptides. Despite shallow sequencing depth, this analysis revealed pMHCs with strong cell type specificity, such as epithelial-derived AGR2 and fibroblast-derived DCN. This cell-level resolution provides biological context that could aid the development of cell-targeted immunotherapies and reduce off-target toxicity.

We also applied PepQueryMHC to re-evaluate the origins of proteasome-spliced peptides previously classified using de novo MS/MS sequencing. Remarkably, transcriptomic evidence was found for 85.71% of the 28 high-confidence peptides, including mappings to non-canonical regions. These findings suggest that a substantial fraction of peptides labeled as cis- or trans-spliced may instead originate from RNA products that were previously unrecognized due to limitations in annotation or mapping strategies. This underscores the need for caution when interpreting spliced peptide annotations and highlights the utility of transcriptomic context in clarifying peptide origin.

Despite its efficiency and versatility, PepQueryMHC, like other RNA-based prioritization tools, is inherently constrained by its reliance on RNA expression as a proxy for peptide presence. This limitation becomes particularly pronounced under conditions of low sequencing depth, such as in single-cell RNA-seq, where sensitivity is significantly reduced. In such contexts, read length can further constrain detection, as the algorithm relies only on reads that fully span a peptide-encoding region. Moreover, while RNA expression may reasonably reflect the presence of peptides derived from protein-coding genes, it fails to capture novel peptides originating from post-transcriptional events, such as ribosomal frameshifts and amino acid substitutions [44, 45]. Ribosome profiling may partially address these limitations, but more advanced technologies are still needed to fully resolve the translational complexity underlying tumor-specific pMHCs. Importantly, even when an RNA “hit” is detected, transcript evidence alone does not ensure antigen presentation. Multiple downstream processes—including translation efficiency, proteasomal cleavage, peptide transport, and HLA loading—collectively determine whether a peptide is ultimately displayed on the cell surface. Therefore, RNA-based matches may yield false positives, where a transcript is present but the peptide is not actually presented, or false negatives, where truly presented peptides are missed due to sequencing depth, read length, or post-translational aberrations. Together, these limitations underscore the need for continued technological and computational innovation to bridge the gap between transcriptomic analyses and actual antigen presentation.

## Conclusions

PepQueryMHC enables rapid and comprehensive integration of pMHC sequences with translated RNA-seq reads, supporting versatile applications including prioritizing class I and II tumor antigens, mapping the cellular origins of presented peptides, and reassessing the prevalence of proteasome-spliced peptides.

## Methods

### Algorithm of PepQueryMHC

PepQueryMHC provides three primary functions: scan mode, target mode, and annotate mode. Both scan mode and target mode count sequencing reads corresponding to given peptides, but they differ in search scope. While scan mode performs an unrestricted search across all reads, target mode restricts the search to specified genomic locations and strands, improving efficiency. Thus, after determining the genomic coordinates and strands of peptides of interest (*e.g.*, tumor antigens) using scan mode, target mode enables focused and rapid expression analysis in other samples. Meanwhile, annotate mode compares peptide-associated genomic information against a reference transcriptome model to classify their transcriptomic annotation categories. Detailed descriptions are provided below.

In the scan mode, the tool takes a list of peptide sequences and a BAM or FASTQ file as input. To efficiently match peptides to reads in the BAM file, the input peptide sequences are indexed using a graph-based structure built with the Aho-Corasick algorithm [25], storing their suffix and prefix relationships (Additional file 1: Fig. S1). The reads in the BAM file are partitioned according to their genomic start positions, allowing parallel processing. Each read is translated into peptide sequences using six-frame or three-frame translation (depending on the strandedness of the sequencing data) and matched against input peptides in linear time, *O(maximum peptide length* + *read length* + *number of matches)*. Unlike BamQuery [20], which relies on the reference genome indices from the STAR aligner [21] and thus missing sample-specific sequences, PepQueryMHC indexes input peptide sequences directly. This approach is significantly more efficient because RNA-seq reads—serving as the search space—are vast and highly variable across samples, and unsuitable for static indexing. Additionally, the complexity of reverse translation increases exponentially with peptide length due to the degeneracy of the genetic code, making direct peptide indexing essential for efficient and scalable peptide-to-transcript mapping, particularly for longer peptides such as MHC-II peptides.

In the target mode, the input includes a list of peptide sequences with their corresponding genomic locations and strands, along with a BAM file. The peptide sequences are distributed across multiple threads for parallel processing. If genomic locations are specified, the tool retrieves reads only from those regions, processes them as in scan mode, and counts reads that exactly match the input peptides.

Annotation mode is designed to consistently classification of peptide types. If no gene is found, the peptide is annotated as an intergenic region (IGR) peptide. Otherwise, transcripts and exon structures are retrieved to classify the peptide into the following categories: in-frame translation (IF), out-of-frame (OOF), non-coding RNA (ncRNA), untranslated region (UTR), intron region (IR), antisense RNA (asRNA), exon skipping (ES) and alternative splicing site (ASS). If multiple genes or transcripts overlap a genomic location, multiple types may be assigned. To address such ambiguities, empirical penalty scores are applied based on prior studies [4, 46]: UTR, OOF = 20, ncRNA = 30, IR = 60, asRNA = 120, IGR = 240, ES = 15 and ASS = 15.

### Datasets of MHC-I- and MHC-II-bound peptides

To evaluate the performance and utility of PepQueryMHC, we downloaded 72,885 class I and 32,597 class II peptides identified from immunopeptidomics profiling of 10 lung adenocarcinoma (LUAD) tumors [5]. Of the 32,597 pMHC-II sequences, 31,526 (96.7%) ranging from 7 to 25 amino acids were selected for analysis. For the evaluation of previously reported cis-/trans-spliced pMHC-I sequences, we obtained four cis-spliced and 24 trans-spliced pMHC-I sequences, for which peptide identification had been validated using synthetic peptides [35] (Additional file 2).

### RNA-seq datasets

For the analysis of LUAD immunopeptidomics data, matched bulk RNA-seq data from ten tumors and their adjacent normal tissues were obtained from Genomic Data Commons (GDC) Data Portal [26]. To evaluate tumor specificity, we obtained 2,403 normal RNA-seq BAM files from healthy tissues through the GTEx consortium (dbGap: phs000424.v8.p2) [18] and eight human mTEC samples from the Gene Expression Omnibus (GEO, GSE127825 and GSE127826) [27, 28]. For cellular origin mapping, single-cell RNA-seq data from eight primary LUAD tumor samples were obtained from the GEO with the accession code GSE123904 [34]. For the analysis of spliced peptides reported by Fradi and colleagues [35], matched RNA-seq data from the B-lymphoblastoid cell lines (C1R) and its monoallelic cell line transfected with HLA-B57:02 (C1R-B57:02) were obtained from the NCBI Sequence Read Archive (SRA) database with the accession code SRP142649 [35]. For the analysis of previously verified spliced peptides [37–41], RNA-seq data of the A-498, A-375 and B-LCL cell lines were obtained from the SRA database under accession codes SRR8616015, SRR8616020 and SRR1925276, respectively [42, 43].

### Bulk RNA-seq analysis

All raw bulk RNA-seq data was aligned to the human genome (GRCh38 primary assembly) using a guided transcript model (GENCODE V38) and processed with the nf_core/rnaseq pipeline v3.14.0 (https://nf-co.re/rnaseq/3.14.0/) [47]. The only modification made was to the STAR aligner, where the parameter “–extra_star_align_args –outSAMunmapped Within” was added to retain unmapped reads; all other settings were kept at their default values. Gviz [48] was used to visualize RNA-seq alignments in detail.

### Single-cell RNA-seq analysis

All single-cell RNA-seq data were processed using the nf_core/scrnaseq pipeline v2.7.1 (https://nf-co.re/scrnaseq/2.7.1) [47], aligning reads to the human genome (GRCh38 primary assembly) with a guided transcript model (GENCODE V38). Cell Ranger was used with the expected number of cells set to 5,000 based on a prior study [34]. Gene expression data were analyzed using the Seurat v5 R package (https://satijalab.org/seurat/articles/seurat5_integration) [49]. Low-quality cells were filtered by selecting those with > 300 transcripts and < 10% mitochondrial transcripts. Gene expression data were then log-normalized using the NormalizeData function with a scale factor of 10,000. Highly variable features were identified using FindVariableFeatures, selecting the top 2,000 most variable genes, followed by data standardization with ScaleData. Principal component analysis (PCA) was conducted using RunPCA to capture key sources of variation. To correct batch effects across samples, we applied canonical correlation analysis (CCA) using IntegrateLayers with CCAIntegration. Cell clustering was performed using FindNeighbors and FindClusters with a resolution of 1, based on the first 30 integrated CCA dimensions, resulting in 21 clusters. Uniform manifold approximation and projection (UMAP) [50] was computed using RunUMAP on the same integrated dimensions (Additional file 1: Fig. S10).

To assign cell types to clusters, we used FindAllMarkers to identify differentially expressed genes (DEGs) for each cluster. Genes with an average fold-change > 2 and an adjusted p-value < 0.01 were considered DEGs. CellMarker 2.0 [51] was utilized as a reference of cell type markers, specifically focusing on marker genes for lung adenocarcinoma and non-small cell lung cancer. The most significantly enriched cell type for each cluster (Fisher’s exact test p-value < 0.05) was assigned accordingly. We further merged detailed cell types (*e.g.*, naïve CD8 + T cell, conventional T cell, regulatory T cell, germinal center B cell, and plasmacytoid dendritic cell) into broader categories (*e.g.*, T cell, B cell, and dendritic cell).

### Selection of cell type-specific peptides

A total of 72,885 class I and 32,597 class II peptides identified from immunopeptidomics profiling of 10 LUAD tumors [5] were queried against single-cell RNA-seq data from eight primary LUAD tumor samples [34] using PepQueryMHC. Based on normalized read counts from PepQueryMHC, the top 10% most abundant peptides were selected for each sample, and the proportion of cells expressing each peptide within each cell type was calculated. Peptides detected in more than half of the cells in at least one cell type were further analyzed using the median-centered coefficient of variation (mCV). To assign a single representative peptide per gene, the highest mCV value among peptides from the same gene was selected as the gene-level mCV. The top 50 genes with the highest mCV values were selected for visualization in Fig. 4b (Additional file 5).

### Comparison with BamQuery

Both PepQueryMHC and BamQuery reported total elapsed runtime, but only PepQueryMHC recorded peak memory usage during computation. To estimate the peak memory usage of BamQuery, we utilized the Python library memory-profiler (v0.61.0) with the options “–include-children –multiprocess” to account for memory consumption across all threads. BamQuery was executed with the following options: “–mode normal –light –t 16 –dbSNP 155 –strandedness”, enabling it to account for known point mutations.

To estimate runtime of BamQuery for longer peptides, we first calculated the total number of coding sequences for each dataset, including subsets of 1 k, 5 k, 10 k, 20 k, 30 k, 40 k 9-mers, as well as the full pMHC-I (8–12-mers) and pMHC-II (7–25-mers) datasets, using Eq. (1):

1 $$Total\;coding\;sequences = \sum\limits_{k}^{n}\prod\limits_{i=1}^{{l}_{k}}C({A}_{k,i})$$

Here, *n* is the total number of peptides, *k* denotes the peptide index, *l*_*k*_ is the length of *peptide*_*k*_, and *A*_*k,i*_ is the amino acid at position *i* in *peptide*_*k*_. *C* is a function that computes the number of codons for a given amino acid (Additional file 1: Fig. S4a). Using the measured runtimes of BamaQuery for 1 k, 5 k, 10 k, 20 k, 30 k, and 40 k 9-mers, we constructed a linear regression model (*runtime* ~ *the number of coding sequences*) (Additional file 1: Fig. S4b). This model was then used to extrapolate runtimes for the full pMHC-I and pMHC-II datasets (Additional file 3). All evaluations were performed on a system with dual Intel Xeon Platinum 8362 CPUs (2.8 GHz, 32 cores) and 1 TB memory.

### Prioritization of tumor antigens

On-target toxicity of tumor antigens can cause severe adverse effects in therapy development and clinical application, necessitating a rigorous prioritization strategy (Additional file 1: Fig. S7a). For 72,885 pMHC-I sequences (8–12-mers) and 31,526 pMHC-II sequences (7–25-mers) in the LUAD dataset [5], we calculated the average RPHM values for each peptide across 2,303 GTEx normal samples (oGTEx), excluding testis and ovary tissues [18]. Since the distributions of average RPHM values varied by peptide length, particularly for MHC-II, we determined and applied length- and type-specific (MHC-I and MHC-II) expression cutoffs at the 1% threshold (Additional file 1: Fig. S7b, c, Additional file 4).

Our multi-step prioritization workflow first filtered peptides based on expression levels, selecting those with maximum RPHM values in LUAD samples exceeding the 1% threshold. To ensure tumor specificity at the RNA level, peptides were excluded if ≥ 1% of oGTEx samples (> 23 samples) had RPHM above the threshold.

Next, peptides were assessed for central immune tolerance, removing those with maximum RPHM values in mTEC or NAT samples exceeding the threshold. The remaining peptides were classified as candidate tumor-specific antigens (TSAs) if fewer than 0.1% of oGTEx samples (< 3 samples) had RPHM values above the threshold. Peptides that did not meet this criterion but had maximum RPHM values in tumor samples exceeding those in oGTEx samples were defined as candidate tumor-associated antigens (TAAs).

To ensure tumor specificity at the protein level, all remaining reference peptides were further filtered using the Human Protein Atlas [16]. Only reference peptides with no detectable protein expression in normal tissues (excluding the testis and ovary) based on immunohistochemistry were retained as TSAs or TAAs, while those with detectable protein expression in these normal tissues were excluded.

Peptides prioritized by our workflow were further evaluated to determine whether they had been previously reported in public immunopeptidome databases, including the Immune Epitope Database (IEDB; 1,069,466 pMHC sequences) [14], IEAtlas (174,465 and 94,375 pMHC sequences from cancer and normal samples, respectively, including non-canonical regions) [13], caAtlas (1,007 cancer-associated antigens and 92 cancer-testis antigens) [15], and the HLA Ligand Atlas (90,428 pMHC-I and 142,625 pMHC-II sequences from 29 benign tissues) [12].

### Systematic evaluation of tumor antigen prioritization

To evaluate the performance of our prioritization strategy, particularly for estimating tumor-specificity, we analyzed 9,172 viral and 223,242 normal pMHCs obtained from the IEDB and the HLA Ligand Atlas, respectively. Three peptides were shared between the two datasets, and these were assigned to the normal pMHC group, resulting in a final count of 9,169 viral pMHCs. The viral pMHCs were retrieved from the IEDB using the following criteria: organism: virus; host: human; epitope structure; linear sequence; assay type: T-cell (positive outcome); disease category: infectious; MHC restriction: any. This ensured the inclusion of T-cell-mediated class I and class II viral epitopes, which were used as the positive dataset. The normal pMHCs were used as the negative dataset. Since our positive data is virus pMHCs, we used 2,403 normal samples in GTEx and 8 mTECs to prioritize virus-specific pMHCs.

### Reannotation of cis- and trans-spliced peptides

We reannotated 28 validated cis-/trans-spliced pMHC-I sequences reported by Fradi and colleagues [35] using PepQueryMHC and BamQuery. For PepQueryMHC, we applied the scan mode followed by annotation mode. Since these peptide sequences were originally derived from de novo peptide sequencing, where isoleucine (I) and leucine (L) could not be distinguished, we treated I/L equivalently during scan mode. In contrast, BamQuery lacks this option, so we enumerated all possible I/L combinations before using them as input.

As a result, PepQueryMHC identified 364 genomic locations, while BamQuery identified 106 that matched the input peptides. After filtering out pMHC-I sequences with RPHM values below the 1% thresholds, 24 peptides remained, spanning 101 genomic locations in PepQueryMHC and 45 in BamQuery. To assign a unique genomic location to each peptide, we prioritized the locations with the highest PRHM value per peptide. Using this finalized set of unique genomic locations and strands, we categorized their transcriptomic annotation categories using the annotate mode in PepQueryMHC for results from both PepQueryMHC and BamQuery (Additional file 1: Fig. S11, Additional file 6).

The PepQueryMHC analysis of the five well-characterized spliced peptides was performed using the same parameters.

## Supplementary Information

Additional file 1: Figures S1-S11.

Additional file 2: Table S1. pMHC datasets used in the performance evaluation.

Additional file 3: Table S2. Results of the performance evaluation.

Additional file 4: Table S3. Prioritized tumor antigens and cutoff RPHM values.

Additional file 5: Table S4. pMHCs of the top 50 genes with the highest median-centered coefficient of variation.

Additional file 6: Table S5. Reannotation of proteasome-spliced peptides.

## Data Availability

All bulk RNA-seq expression datasets of MHC-I and MHC-II sequences generated by PepQueryMHC, along with the Seurat object containing the processed single-cell RNA-seq dataset from eight primary lung adenocarcinoma samples, are available under the GNU General Public License v3.0 at 10.5281/zenodo.17429717 [52]. PepQueryMHC was implemented in Java (open-jdk 21 or higher, platform independent). htsjdk (version 4.1.0) was used to process BAM/FASTQ files and ahocorasick (version 0.6.3) was used to index peptides and search reads in a BAM/FASTQ file. PepQueryMHC is available under the GNU General Public License v3.0 at https://github.com/bzhanglab/PepQueryMHC [53], and all data analysis code used in this study is also available at the same repository. The version of source code used in the manuscript is also deposited in 10.5281/zenodo.17429717 [52]. Previously identified MHC-I and MHC-II sequences from LUAD tumors were obtained from a published study [5]. Matched bulk RNA-seq datasets from ten LUAD and their adjacent normal tissues were obtained from the GDC Data Portal (https://portal.gdc.cancer.gov) under dbGaP Study Accession phs001287.v5.p4 [26, 54]. Normal tissue RNA-seq datasets comprising 2,403 BAM files were obtained from the GTEx consortium under dbGap accession phs000424.v8.p2 [18, 55]. Eight human mTEC RNA-seq datasets were downloaded from GEO under accessions GSE127825 and GSE127826 [27, 28, 56, 57]. For cellular origin mapping, single-cell RNA-seq datasets from eight primary LUAD samples were obtained from GEO under accession GSE123904 [34, 58]. For the analysis of spliced peptides reported by Fradi et al. [35], matched RNA-seq datasets from C1R and C1R-B57:02 cell lines were obtained from the NCBI SRA database with the accession code SRP142649 [35, 59]. Previously validated spliced peptides [37–41] were analyzed using RNA-seq datasets from the A-498, A-375 and B-LCL cell lines obtained from the NCBI SRA under accessions SRR8616015, SRR8616020 and SRR1925276, respectively [42, 43, 60–62].

## References

[1] Pishesha N, Harmand TJ, Ploegh HL. A guide to antigen processing and presentation. Nat Rev Immunol. 2022;22:751–64. 35418563 10.1038/s41577-022-00707-2

[2] Xie N, Shen G, Gao W, Huang Z, Huang C, Fu L. Neoantigens: promising targets for cancer therapy. Signal Transduct Target Ther. 2023;8:9. 36604431 10.1038/s41392-022-01270-x PMC9816309

[3] Pan Y, Kadash-Edmondson KE, Wang R, Phillips J, Liu S, Ribas A, et al. RNA dysregulation: an expanding source of cancer immunotherapy targets. Trends Pharmacol Sci. 2021;42:268–82. 33711255 10.1016/j.tips.2021.01.006 PMC8761020

[4] Choi S, Paek E. Pxg: comprehensive identification of noncanonical MHC-I-associated peptides from de novo peptide sequencing using RNA-seq reads. Mol Cell Proteomics. 2024;23:100743. 38403075 10.1016/j.mcpro.2024.100743 PMC10979277

[5] Abelin JG, Bergstrom EJ, Rivera KD, Taylor HB, Klaeger S, Xu C, et al. Workflow enabling deepscale immunopeptidome, proteome, ubiquitylome, phosphoproteome, and acetylome analyses of sample-limited tissues. Nat Commun. 2023;14:1851. 37012232 10.1038/s41467-023-37547-0 PMC10070353

[6] Chong C, Coukos G, Bassani-Sternberg M. Identification of tumor antigens with immunopeptidomics. Nat Biotechnol. 2022;40:175–88. 34635837 10.1038/s41587-021-01038-8

[7] Huber F, Arnaud M, Stevenson BJ, Michaux J, Benedetti F, Thevenet J, et al. A comprehensive proteogenomic pipeline for neoantigen discovery to advance personalized cancer immunotherapy. Nat Biotechnol. 2024. 10.1038/s41587-024-02420-y. 39394480 10.1038/s41587-024-02420-y PMC12339364

[8] Zhang B, Bassani-Sternberg M. Current perspectives on mass spectrometry-based immunopeptidomics: the computational angle to tumor antigen discovery. J Immunother Cancer. 2023;11:e007073. 37899131 10.1136/jitc-2023-007073 PMC10619091

[9] Hundal J, Carreno BM, Petti AA, Linette GP, Griffith OL, Mardis ER, et al. Pvac-seq: a genome-guided in silico approach to identifying tumor neoantigens. Genome Med. 2016;8:11. 26825632 10.1186/s13073-016-0264-5 PMC4733280

[10] Zhou C, Wei Z, Zhang Z, Zhang B, Zhu C, Chen K, et al. Ptuneos: prioritizing tumor neoantigens from next-generation sequencing data. Genome Med. 2019;11:67. 31666118 10.1186/s13073-019-0679-x PMC6822339

[11] Hundal J, Kiwala S, McMichael J, Miller CA, Xia H, Wollam AT, et al. PVactools: a computational toolkit to identify and visualize cancer neoantigens. Cancer Immunol Res. 2020;8:409–20. 31907209 10.1158/2326-6066.CIR-19-0401 PMC7056579

[12] Marcu A, Bichmann L, Kuchenbecker L, Kowalewski DJ, Freudenmann LK, Backert L, et al. HLA ligand atlas: a benign reference of HLA-presented peptides to improve T-cell-based cancer immunotherapy. J Immunother Cancer. 2021;9:e002071. 33858848 10.1136/jitc-2020-002071 PMC8054196

[13] Cai Y, Lv D, Li D, Yin J, Ma Y, Luo Y, et al. IEAtlas: an atlas of HLA-presented immune epitopes derived from non-coding regions. Nucleic Acids Res. 2023;51:D409–17. 36099422 10.1093/nar/gkac776 PMC9825419

[14] Vita R, Blazeska N, Marrama D, IEDB Curation Team Members, Duesing S, Bennett J, et al. The Immune Epitope Database (IEDB): 2024 update. Nucleic Acids Res. 2025;53:D436–43. 10.1093/nar/gkae1092 PMC11701597 39558162

[15] Yi X, Liao Y, Wen B, Li K, Dou Y, Savage SR, et al. Caatlas: an immunopeptidome atlas of human cancer. iScience. 2021;24:103107. 34622160 10.1016/j.isci.2021.103107 PMC8479791

[16] Uhlén M, Fagerberg L, Hallström BM, Lindskog C, Oksvold P, Mardinoglu A, et al. Tissue-based map of the human proteome. Science. 2015;347:1260419. 25613900 10.1126/science.1260419

[17] Cancer Genome Atlas Research Network, Weinstein JN, Collisson EA, Mills GB, Shaw KRM, Ozenberger BA, et al. The cancer genome atlas pan-cancer analysis project. Nat Genet. 2013;45:1113–20. 24071849 10.1038/ng.2764 PMC3919969

[18] GTEx Consortium. The genotype-tissue expression (GTEx) project. Nat Genet. 2013;45:580–5. 23715323 10.1038/ng.2653 PMC4010069

[19] Yi X, Zhao H, Hu S, Dong L, Dou Y, Li J, Gao Q, Zhang B. Tumor-associated antigen prediction using a single-sample gene expression state inference algorithm. Cell Rep Methods. 2024;4(11):100906. 10.1016/j.crmeth.2024.100906. PMID: 39561714; PMCID: PMC11705763. 10.1016/j.crmeth.2024.100906 PMC11705763 39561714

[20] Cuevas MVR, Hardy M-P, Larouche J-D, Apavaloaei A, Kina E, Vincent K, et al. Bamquery: a proteogenomic tool to explore the immunopeptidome and prioritize actionable tumor antigens. Genome Biol. 2023;24:188. 37582761 10.1186/s13059-023-03029-1 PMC10426134

[21] Dobin A, Davis CA, Schlesinger F, Drenkow J, Zaleski C, Jha S, et al. STAR: ultrafast universal RNA-seq aligner. Bioinformatics. 2013;29:15–21. 23104886 10.1093/bioinformatics/bts635 PMC3530905

[22] Apavaloaei A, Zhao Q, Hesnard L, Cahuzac M, Durette C, Larouche J-D, et al. Tumor antigens preferentially derive from unmutated genomic sequences in melanoma and non-small cell lung cancer. Nat Cancer. 2025. 10.1038/s43018-025-00979-2. 40405018 10.1038/s43018-025-00979-2 PMC12380612

[23] Askenazi M, Ruggles KV, Fenyö D. PGx: putting peptides to BED. J Proteome Res. 2016;15:795–9. 26638927 10.1021/acs.jproteome.5b00870 PMC4782174

[24] Choi S, Kim H, Paek E. ACTG: novel peptide mapping onto gene models. Bioinformatics. 2017;33:1218–20. 28031186 10.1093/bioinformatics/btw787

[25] Aho AV, Corasick MJ. Efficient string matching: an aid to bibliographic search. Commun ACM. 1975;18:333–40.

[26] Gillette MA, Satpathy S, Cao S, Dhanasekaran SM, Vasaikar SV, Krug K, et al. Proteogenomic characterization reveals therapeutic vulnerabilities in lung adenocarcinoma. Cell. 2020;182:200-25.e35. 32649874 10.1016/j.cell.2020.06.013 PMC7373300

[27] Laumont CM, Vincent K, Hesnard L, Audemard É, Bonneil É, Laverdure J-P, et al. Noncoding regions are the main source of targetable tumor-specific antigens. Sci Transl Med. 2018;10:eaau5516. 30518613 10.1126/scitranslmed.aau5516

[28] Larouche J-D, Trofimov A, Hesnard L, Ehx G, Zhao Q, Vincent K, et al. Widespread and tissue-specific expression of endogenous retroelements in human somatic tissues. Genome Med. 2020;12:40. 32345368 10.1186/s13073-020-00740-7 PMC7189544

[29] Cameron BJ, Gerry AB, Dukes J, Harper JV, Kannan V, Bianchi FC, et al. Identification of a Titin-derived HLA-A1-presented peptide as a cross-reactive target for engineered MAGE A3-directed T cells. Sci Transl Med. 2013;5:197ra103. 23926201 10.1126/scitranslmed.3006034 PMC6002776

[30] Scanlan MJ, Altorki NK, Gure AO, Williamson B, Jungbluth A, Chen YT, et al. Expression of cancer-testis antigens in lung cancer: definition of bromodomain testis-specific gene (BRDT) as a new CT gene, CT9. Cancer Lett. 2000;150:155–64. 10704737 10.1016/s0304-3835(99)00385-7

[31] Mobasheri MB, Shirkoohi R, Zendehdel K, Jahanzad I, Talebi S, Afsharpad M, et al. Transcriptome analysis of the cancer/testis genes, DAZ1, AURKC, and TEX101, in breast tumors and six breast cancer cell lines. Tumour Biol. 2015;36:8201–6. 25994570 10.1007/s13277-015-3546-4

[32] Müller S, Bley N, Glaß M, Busch B, Rousseau V, Misiak D, et al. IGF2BP1 enhances an aggressive tumor cell phenotype by impairing miRNA-directed downregulation of oncogenic factors. Nucleic Acids Res. 2018;46:6285–303. 29660014 10.1093/nar/gky229 PMC6158595

[33] Müller S, Bley N, Busch B, Glaß M, Lederer M, Misiak C, et al. The oncofetal RNA-binding protein IGF2BP1 is a druggable, post-transcriptional super-enhancer of E2F-driven gene expression in cancer. Nucleic Acids Res. 2020;48:8576–90. 32761127 10.1093/nar/gkaa653 PMC7470957

[34] Laughney AM, Hu J, Campbell NR, Bakhoum SF, Setty M, Lavallée V-P, et al. Regenerative lineages and immune-mediated pruning in lung cancer metastasis. Nat Med. 2020;26:259–69. 32042191 10.1038/s41591-019-0750-6 PMC7021003

[35] Faridi P, Li C, Ramarathinam SH, Vivian JP, Illing PT, Mifsud NA, et al. A subset of HLA-I peptides are not genomically templated: evidence for cis- and trans-spliced peptide ligands. Sci Immunol. 2018;3:eaar3947. 30315122 10.1126/sciimmunol.aar3947

[36] Admon A. Are there indeed spliced peptides in the immunopeptidome? Mol Cell Proteomics. 2021;20:100099. 34022431 10.1016/j.mcpro.2021.100099 PMC8724635

[37] Hanada K-I, Yewdell JW, Yang JC. Immune recognition of a human renal cancer antigen through post-translational protein splicing. Nature. 2004;427:252–6. 14724640 10.1038/nature02240

[38] Vigneron N, Stroobant V, Chapiro J, Ooms A, Degiovanni G, Morel S, et al. An antigenic peptide produced by peptide splicing in the proteasome. Science. 2004;304:587–90. 15001714 10.1126/science.1095522

[39] Michaux A, Larrieu P, Stroobant V, Fonteneau J-F, Jotereau F, den Van Eynde BJ, et al. A spliced antigenic peptide comprising a single spliced amino acid is produced in the proteasome by reverse splicing of a longer peptide fragment followed by trimming. J Immunol. 2014;192:1962–71. 24453253 10.4049/jimmunol.1302032

[40] Warren EH, Vigneron NJ, Gavin MA, Coulie PG, Stroobant V, Dalet A, et al. An antigen produced by splicing of noncontiguous peptides in the reverse order. Science. 2006;313:1444–7. 16960008 10.1126/science.1130660

[41] Dalet A, Robbins PF, Stroobant V, Vigneron N, Li YF, El-Gamil M, et al. An antigenic peptide produced by reverse splicing and double asparagine deamidation. Proc Natl Acad Sci U S A. 2011;108:E323-31. 21670269 10.1073/pnas.1101892108 PMC3142003

[42] Barretina J, Caponigro G, Stransky N, Venkatesan K, Margolin AA, Kim S, et al. The cancer cell line encyclopedia enables predictive modelling of anticancer drug sensitivity. Nature. 2012;483:603–7. 22460905 10.1038/nature11003 PMC3320027

[43] Laumont CM, Daouda T, Laverdure J-P, Bonneil É, Caron-Lizotte O, Hardy M-P, et al. Global proteogenomic analysis of human MHC class I-associated peptides derived from non-canonical reading frames. Nat Commun. 2016;7:10238. 26728094 10.1038/ncomms10238 PMC4728431

[44] Bartok O, Pataskar A, Nagel R, Laos M, Goldfarb E, Hayoun D, et al. Anti-tumour immunity induces aberrant peptide presentation in melanoma. Nature. 2021;590:332–7. 33328638 10.1038/s41586-020-03054-1

[45] Pataskar A, Champagne J, Nagel R, Kenski J, Laos M, Michaux J, et al. Tryptophan depletion results in tryptophan-to-phenylalanine substitutants. Nature. 2022;603:721–7. 35264796 10.1038/s41586-022-04499-2 PMC8942854

[46] Erhard F, Dölken L, Schilling B, Schlosser A. Identification of the cryptic HLA-I immunopeptidome. Cancer Immunol Res. 2020;8:1018–26. 32561536 10.1158/2326-6066.CIR-19-0886

[47] Ewels PA, Peltzer A, Fillinger S, Patel H, Alneberg J, Wilm A, et al. The nf-core framework for community-curated bioinformatics pipelines. Nat Biotechnol. 2020;38:276–8. 32055031 10.1038/s41587-020-0439-x

[48] Hahne F, Ivanek R. Visualizing genomic data using Gviz and Bioconductor. Methods Mol Biol. 2016;1418:335–51. 27008022 10.1007/978-1-4939-3578-9_16

[49] Butler A, Hoffman P, Smibert P, Papalexi E, Satija R. Integrating single-cell transcriptomic data across different conditions, technologies, and species. Nat Biotechnol. 2018;36:411–20. 29608179 10.1038/nbt.4096 PMC6700744

[50] McInnes L, Healy J, Melville J. UMAP: Uniform Manifold Approximation and Projection for Dimension Reduction. arXiv [stat.ML]. 2018. Available from: http://arxiv.org/abs/1802.03426

[51] Hu C, Li T, Xu Y, Zhang X, Li F, Bai J, et al. CellMarker 2.0: an updated database of manually curated cell markers in human/mouse and web tools based on scRNA-seq data. Nucleic Acids Res. 2023;51:D870–6. 10.1093/nar/gkac947 PMC9825416 36300619

[52] Choi S, Zhang B. PepQueryMHC: Rapid and comprehensive tumor antigen prioritization from immunopeptidomics data. Zenodo. https://zenodo.org/records/17429717 (2025). 10.1186/s13059-025-03923-w PMC12723928 41437377

[53] Choi S, Zhang B. PepQueryMHC. GitHub. https://github.com/bzhanglab/PepQueryMHC (2025).

[54] CPTAC Consortium. CPTAC 3 Study. dbGaP. https://identifiers.org/dbGaP:phs001287.v5.p4. (2020).

[55] GTEx Consortium. Common Fund (CF) Genotype-Tissue Expression Project (GTEx). dbGaP. https://identifiers.org/dbGaP:phs000424.v8.p2. (2013).

[56] Laumont CM, Vincent K, Hesnard L, Audemard É, Bonneil É, Laverdure JP, et al. Noncoding regions are the main source of targetable tumor-specific antigens. Sci Transl Med. 2018. 10.1126/scitranslmed.aau5516. 30518613 10.1126/scitranslmed.aau5516

[57] Larouche JD, Trofimov A, Hesnard L, Ehx G, Zhao Q, Vincent K, Durette C, Gendron P, Laverdure JP, Bonneil É, Côté C, Lemieux S, Thibault P, Perreault C. Complementary transcriptomic analysis of human medullary thymic epithelial cells. Gene Expression Omnibus. https://identifiers.org/geo:GSE127826. (2019).

[58] Laughney AM, Hu J, Campbell NR, Bakhoum SF, Setty M, Lavallée V-P, et al.The single cell transcriptional landscape of lung adenocarcinoma metastasis. Gene Expression Omnibus. https://identifiers.org/geo:GSE123904. (2020).

[59] Faridi P, Li C, Ramarathinam SH, Vivian JP, Illing PT, Mifsud NA, et al. RNA-Seq of Homo sapiens lymphoblastoid cell line. NCBI Sequence Read Archive. https://www.ncbi.nlm.nih.gov/sra/?term=SRP142649. (2018).

[60] Barretina J, Caponigro G, Stransky N, Venkatesan K, Margolin AA, Kim S, et al. RNAseq of A498_KIDNEY. NCBI Sequence Read Archive. https://www.ncbi.nlm.nih.gov/sra/?term=SRR8616015. (2019).

[61] Barretina J, Caponigro G, Stransky N, Venkatesan K, Margolin AA, Kim S, et al. RNAseq of A375_SKIN. NCBI Sequence Read Archive. https://www.ncbi.nlm.nih.gov/sra/?term=SRR8616020. (2019).

[62] Laumont CM, Daouda T, Laverdure J-P, Bonneil É, Caron-Lizotte O, Hardy M-P, et al. GSM1641204: B-LCL-2.1 [reanalysis]; Homo sapiens; RNA-seq. NCBI Sequence Read Archive. https://www.ncbi.nlm.nih.gov/sra/?term=SRR1925276. (2016).

