# Supplementary material for: PepQueryMHC: rapid and comprehensive tumor antigen prioritization from immunopeptidomics data
Source: Genome Biol. 2025 Dec 23;26:434. doi: 10.1186/s13059-025-03923-w (PMC12723928; doi:10.1186/s13059-025-03923-w)
Supplement: Supplementary file 1 — Additional file 1: Figures S1-S11. [file 13059_2025_3923_MOESM1_ESM.docx]

**PepQueryMHC: Rapid and comprehensive tumor antigen prioritization from immunopeptidomics data**

Seunghyuk Choi^1,2,3^ and Bing Zhang^1,2*^

^1^Lester and Sue Smith Breast Center, Baylor College of Medicine, Houston, TX 77030, USA

^2^Department of Molecular and Human Genetics, Baylor College of Medicine, Houston, TX 77030, USA

^3^School of Software, College of Computer Science, Kookmin University, Seoul 02707, Republic of Korea

Email: Seunghyuk Choi {shchoi8@kookmin.ac.kr}, Bing Zhang {bing.zhang@bcm.edu}

***To whom correspondence should be addressed:**

Bing Zhang

Lester and Sue Smith Breast Center, Baylor College of Medicine

1 Baylor Plaza, Houston, TX 77030, USA

Tel: +1 (713) 798-1443

Email: bing.zhang@bcm.edu


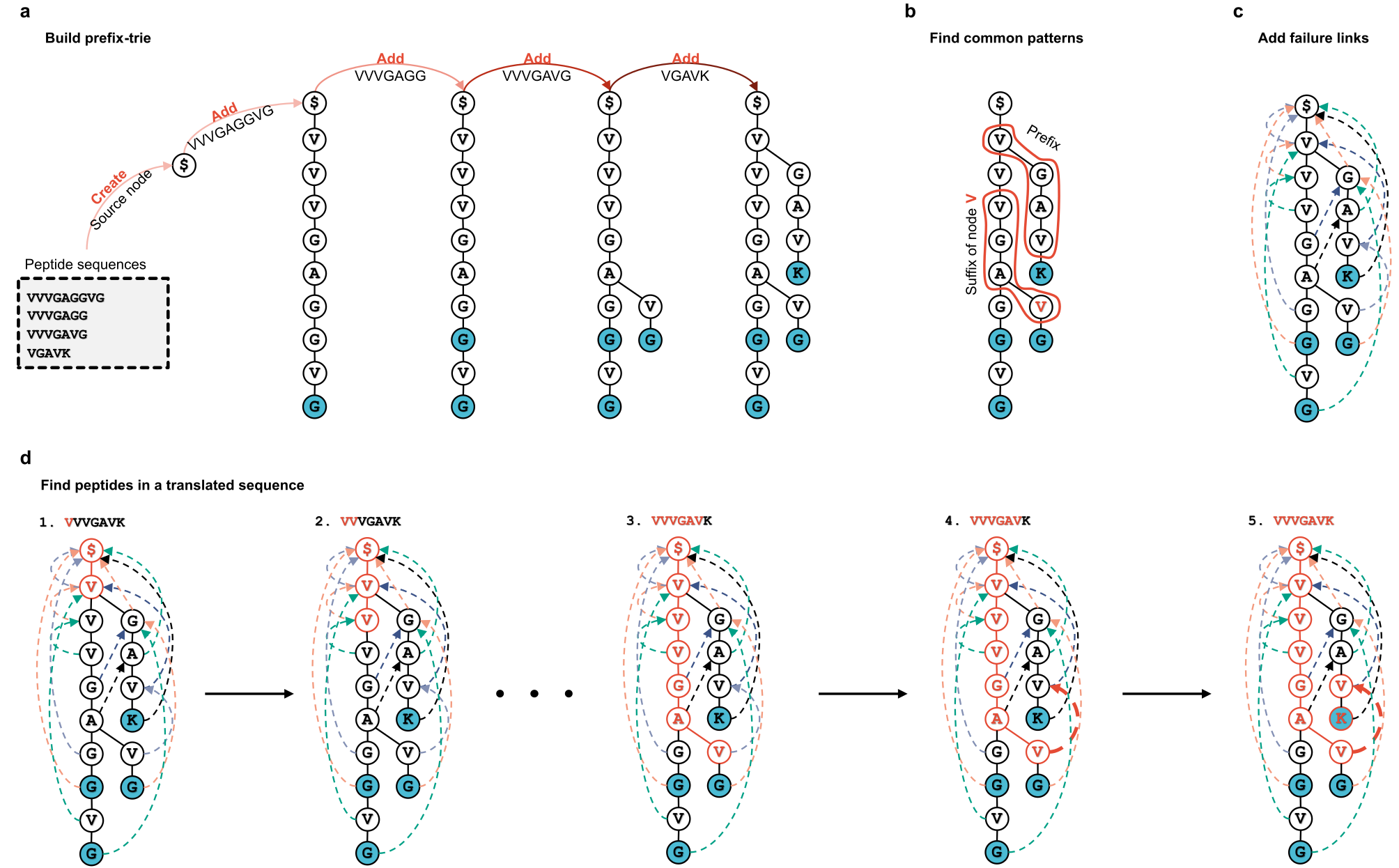


**Fig. S1** Multiple pattern matching algorithm. **a** A prefix-trie is constructed from the input peptide sequences by sequentially adding each sequence. **b** An example of a common pattern between the suffix of a given node V and any prefix. **c** Using these common patterns, each node establishes a failure link that links connects nodes with a suffix-prefix relationship. **d** An example demonstrating peptide search within a translated sequence. The trie is traversed sequentially as the translated sequence is read amino acid by amino acid. If a node matches the current amino acid, the traversal moves to that node (step 1-4). If no match is found, it follows the failure link and continues traversal from the linked node (step 5).


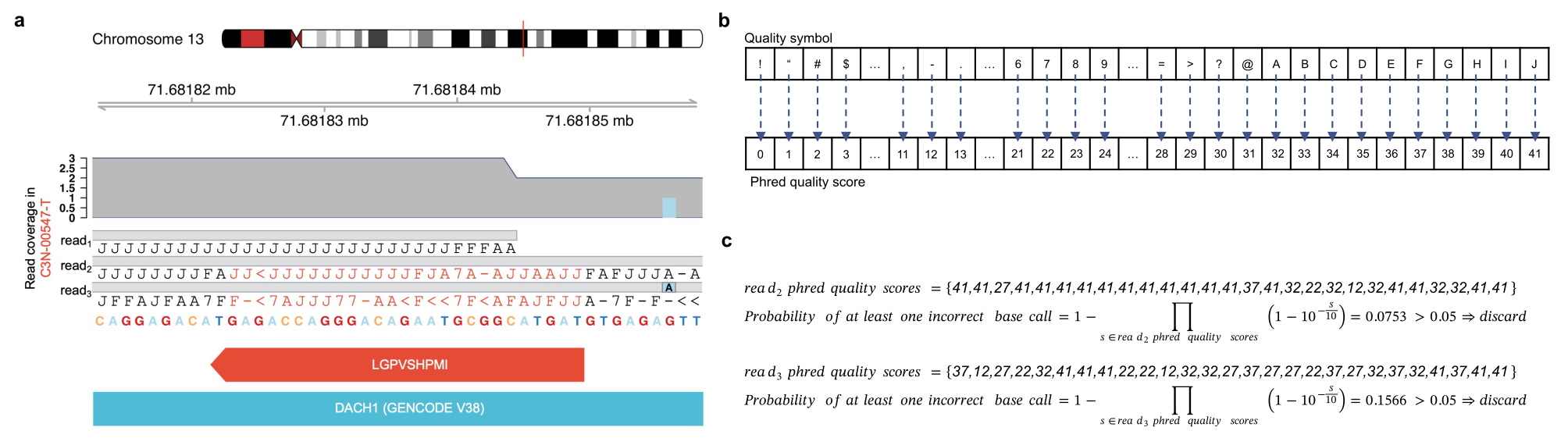


**Fig. S2** Example of filtering low quality reads. **a** Each read contains sequencing quality symbols (e.g., J, F, A). Only the qualities in the region matched to the peptide are considered. **b** Each symbol is converted into a Phred quality score. **e,** Illustration of how to calculate the probability of at least one incorrect base call for read_2_ and read_3_.


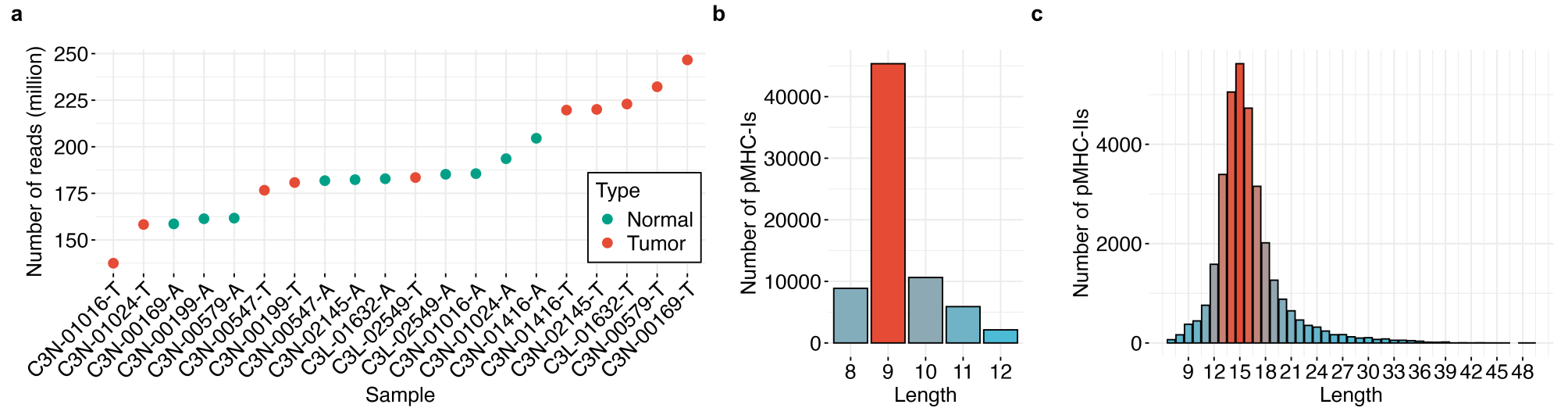


**Fig. S3** Evaluation data overview. **a** RNA-seq read counts across 10 lung adenocarcinoma tumors and their paired adjacent normal tissues. **b, c** Length distributions of pMHC-I sequences (**b**) and pMHC-II sequences (**c**) identified by immunopeptidomics in the 10 tumor samples.


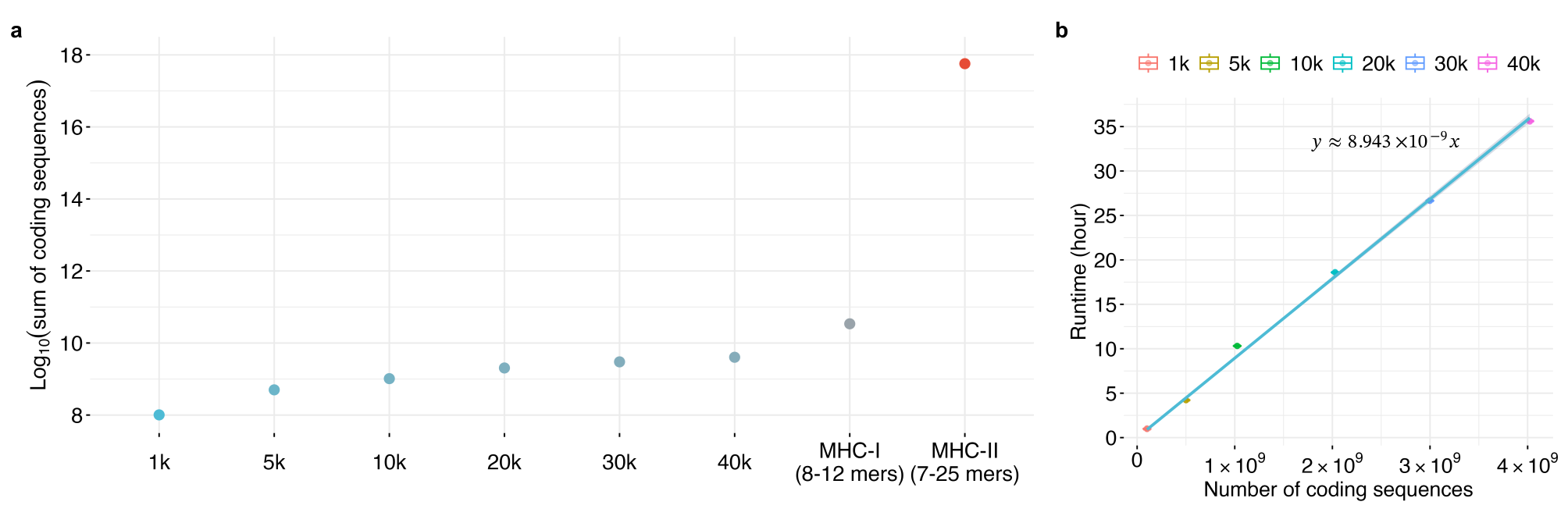


**Fig. S4** Linear regression model for predicting BamQuery runtimes. **a** Total number of coding sequences generated by reverse-translating varying numbers of peptides, including 1k, 5k, 10k, 20k, 30k, and 40k randomly sampled pMHC-I 9-mers, as well as all MHC-I and II peptides in the LUAD datasets. **b** BamQuery runtimes were measured three times and modeled using linear regression based on the number of coding sequences.


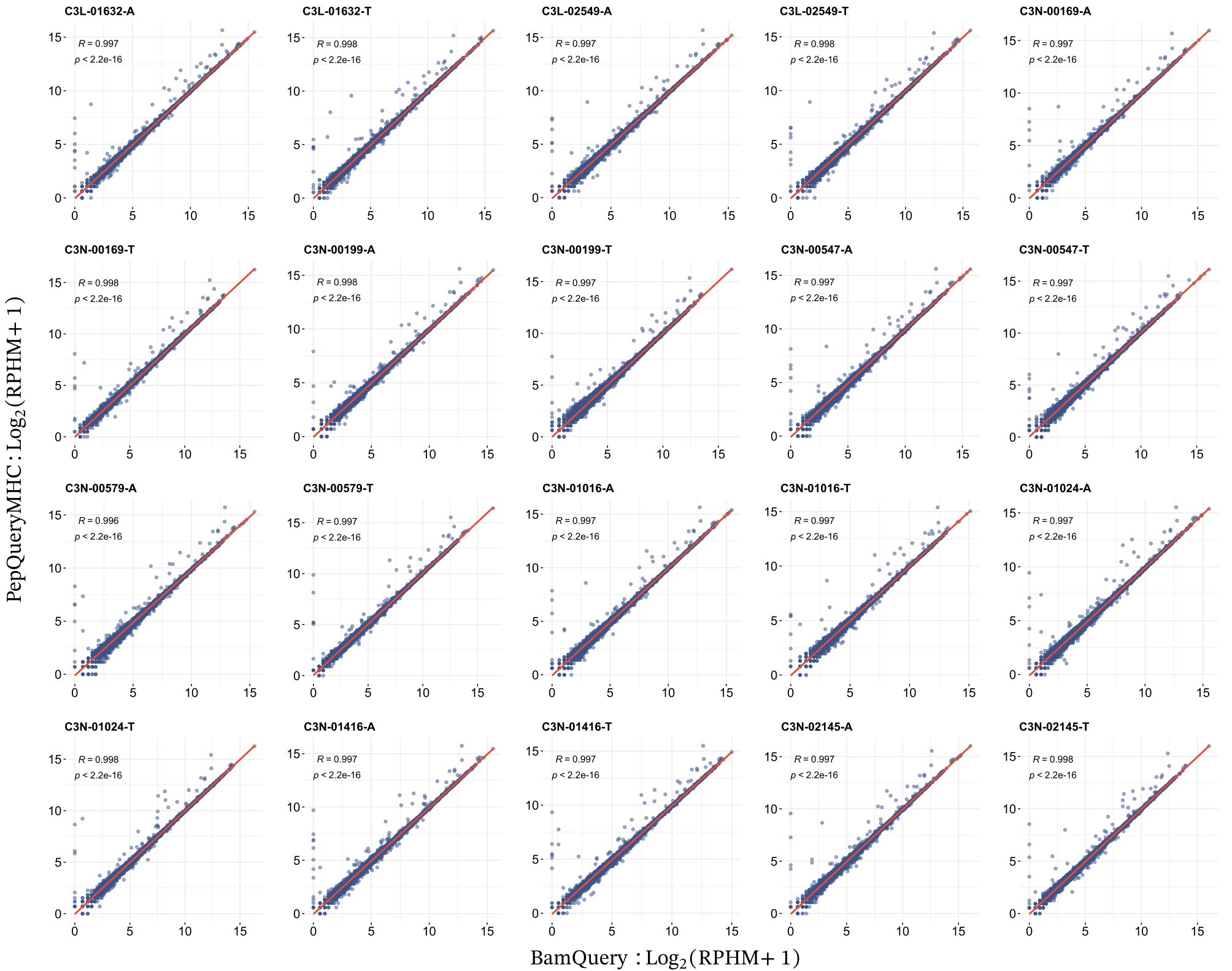


**Fig. S5** Scatterplots comparing RPHMs between PepQueryMHC and BamQuery quantifications. Pearson’s correlation coefficients (R) were calculated using 40k 9-mers across 20 lung adenocarcinoma samples.


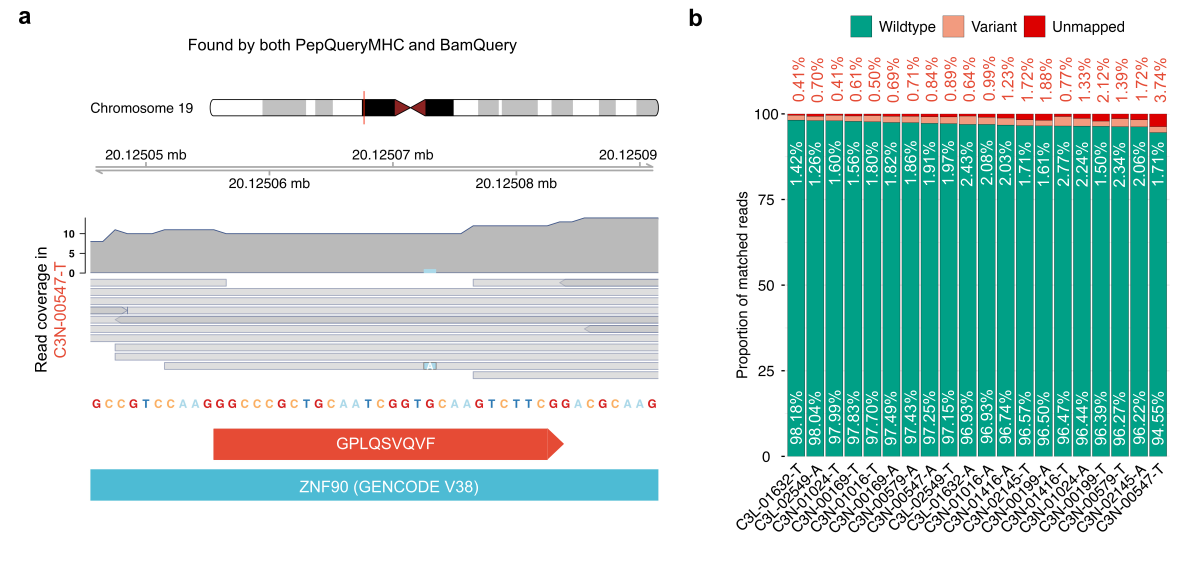


**Fig. S6** Inconsistent read counts between PepQueryMHC and BamQuery. **a** Peptide “GPLQSVQVF” aligns to regions in both ZNF90 and RPS16 genes. PepQueryMHC exclusively matches reads in RPS16 by accounting for two synonymous point mutations within the region. **b** Proportion of reads matching pMHC sequences in the 40k 9-mers dataset analyzed using PepQueryMHC. Matched reads that do not align to the genome are categorized as “Unmapped.” Among the remaining reads, those containing sequences variants are labeled as “Variant,” while those without variants are classified as “Wildtype.”


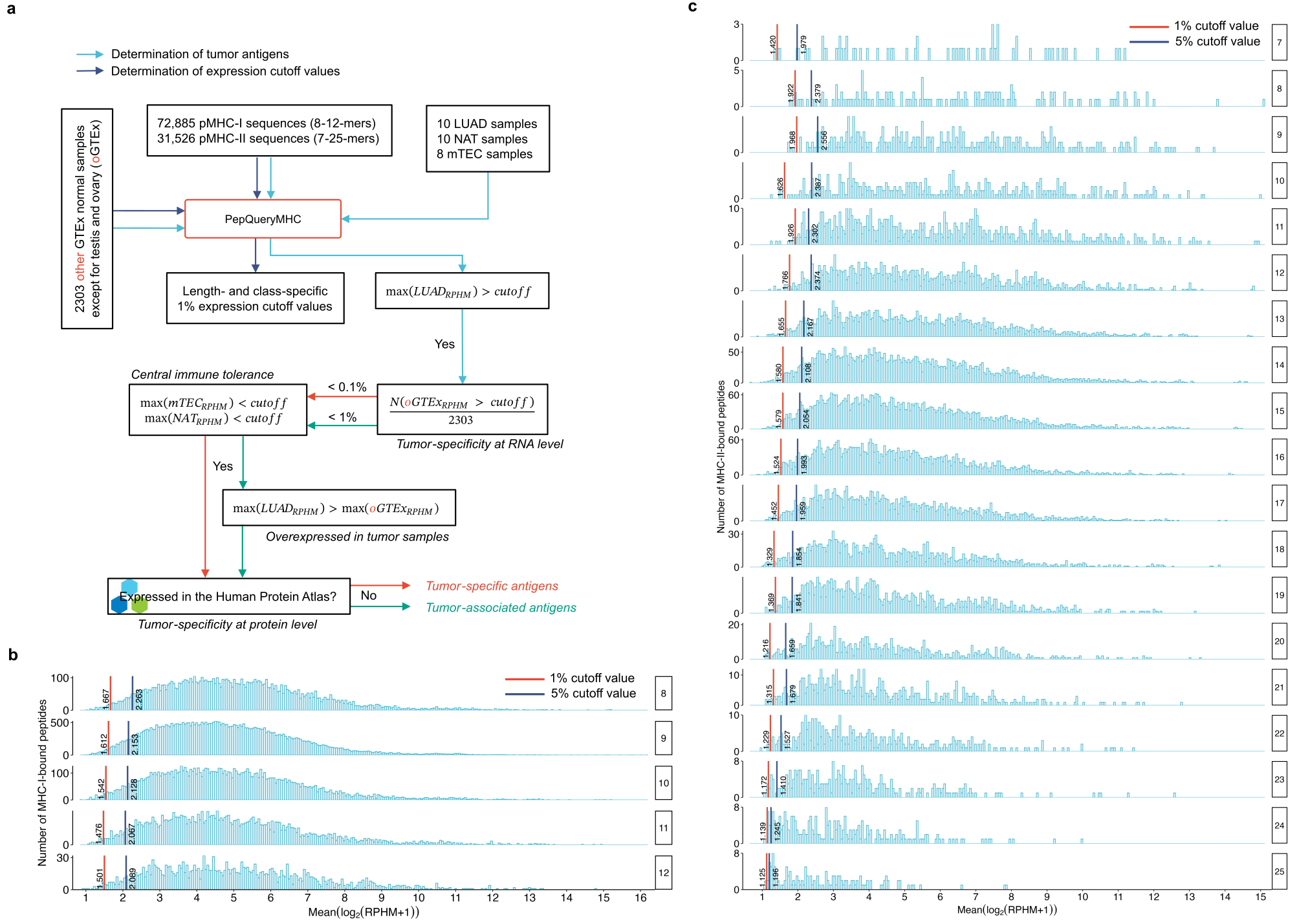


**Fig. S7** Rigorous strategy for prioritizing tumor antigens. **a** Overall description of steps accounting for tumor-specificity at RNA level, central immune tolerance, overexpressed tumor antigens and tumor-specificity at protein level. **b, c** Distributions of average RPHM values for each peptide length in MHC-I (**b**) and MHC-II (**c**).


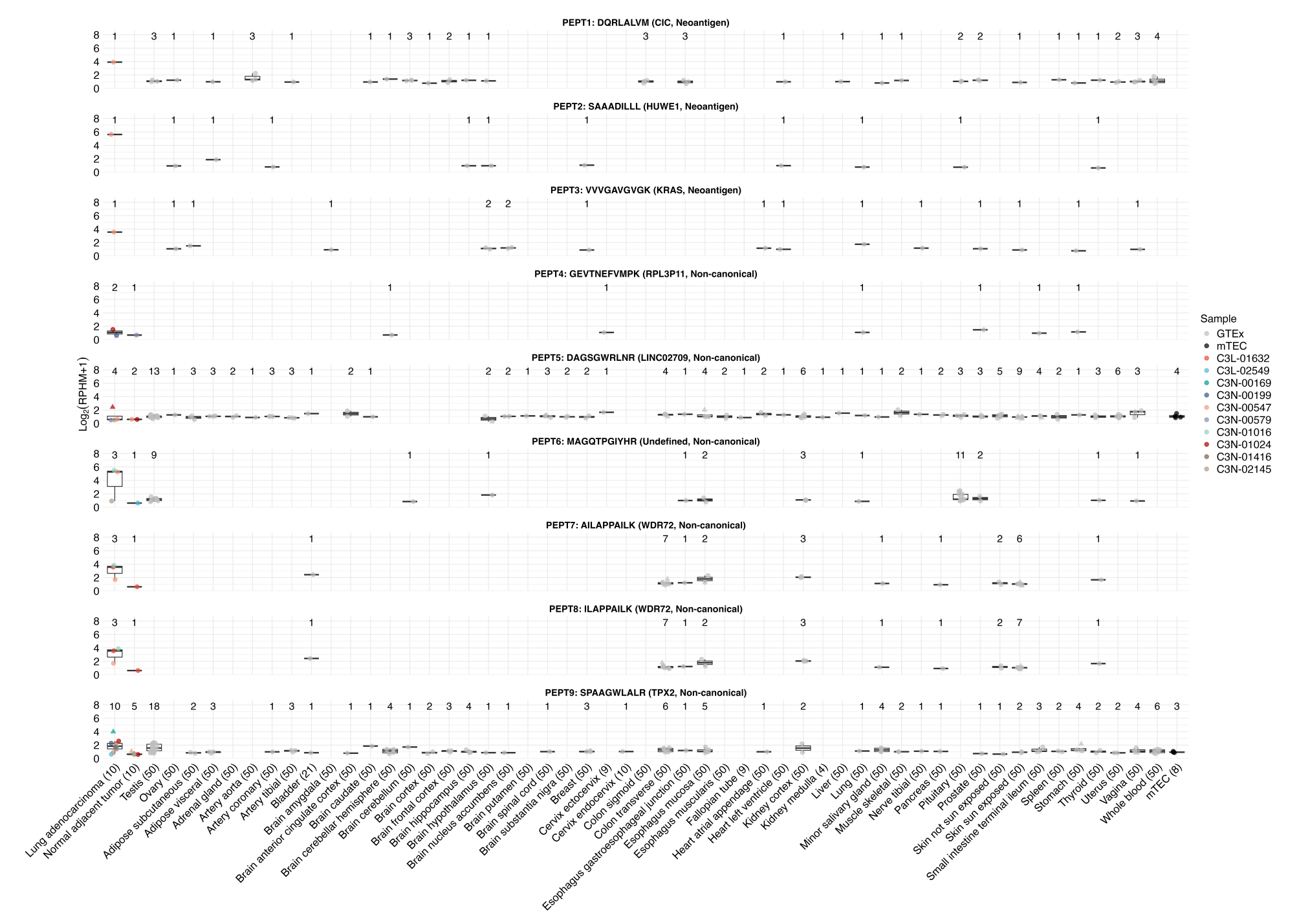


**Fig. S8** RPHM distribution of the non-reference antigen peptides across samples. Boxplots display RPHM values across samples for individual peptides. Outliers (below Q1 – 1.5 x IQR or above Q3 + 1.5 x IQR) are presented by triangles.


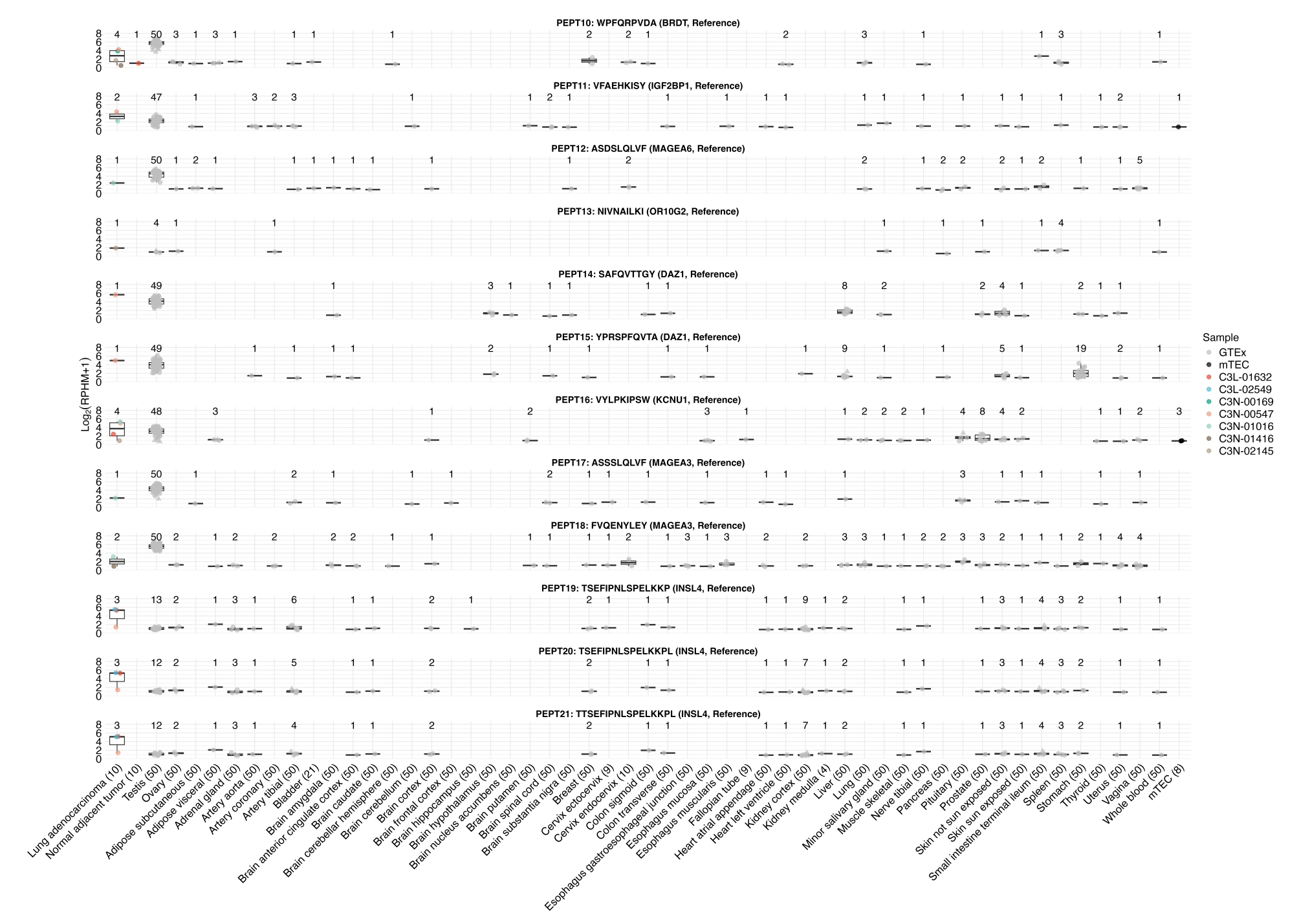


**Fig. S9** RPHM distribution of the reference antigen peptides across samples. Boxplots display RPHM values across samples for individual peptides. Outliers (below Q1 – 1.5 x IQR or above Q3 + 1.5 x IQR) are presented by triangles.


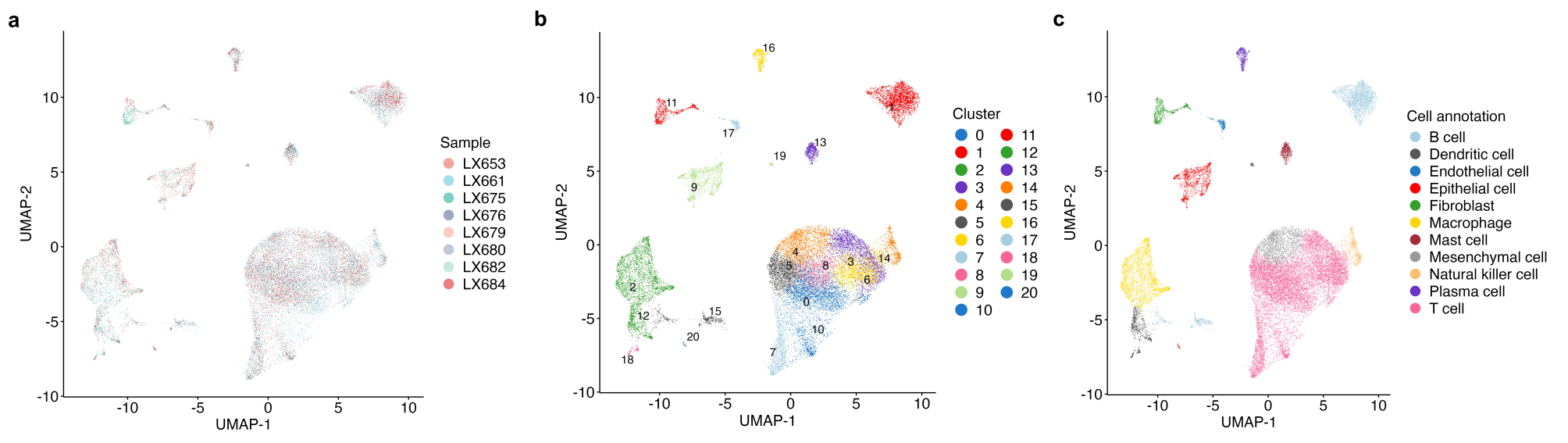


**Fig. S10** UMAP projection of all cells from eight primary LUAD tumors in a single-cell RNA-seq dataset. **a-c** The UMAP is colored by sample name (**a**), sample clusters (**b**), and cell types (**c**), respectively.


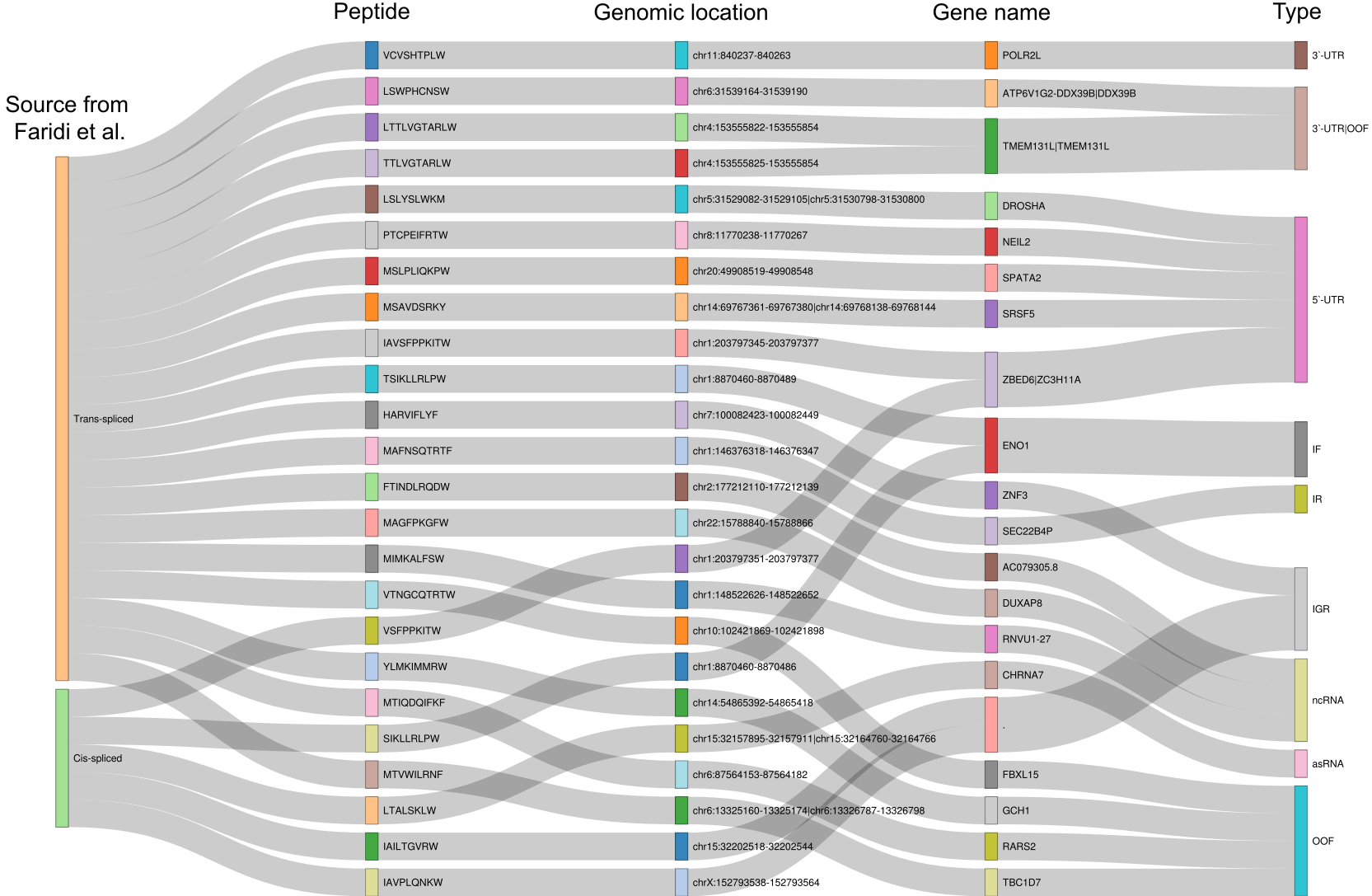


**Fig. S11** Reannotation of previously reported cis- and trans-spliced pMHC-I sequences. PepQueryMHC mapped 24 previously reported cis- and trans-spliced pMHC-I sequences to RNA-seq reads from matched samples. The new annotations include nine categories: in-frame translation (IF), out-of-frame (OOF), 5`- or 3`-untranslated region (5`- or 3`-UTR), non-coding RNA (ncRNA), intron retention (IR), antisense RNA (asRNA), and intergenic region (IGR). The vertical bar (|) indicates “or.”
